# Future perspectives in melanoma research. Meeting report from the “Melanoma Bridge. Napoli, December 2nd-4th 2012”

**DOI:** 10.1186/1479-5876-11-137

**Published:** 2013-06-03

**Authors:** Paolo A Ascierto, Antonio M Grimaldi, Nicolas Acquavella, Lorenzo Borgognoni, Luana Calabrò, Natale Cascinelli, Alessandra Cesano, Michele Del Vecchio, Alexander M Eggermont, Mark Faries, Soldano Ferrone, Bernard A Fox, Thomas F Gajewski, Jérôme Galon, Sacha Gnjatic, Helen Gogas, Mohammed Kashani-Sabet, Howard L Kaufman, James Larkin, Roger S Lo, Alberto Mantovani, Kim Margolin, Cornelis Melief, Grant McArthur, Giuseppe Palmieri, Igor Puzanov, Antoni Ribas, Barbara Seliger, Jeff Sosman, Peter Suenaert, Ahmad A Tarhini, Giorgio Trinchieri, Fernando Vidal-Vanaclocha, Ena Wang, Gennaro Ciliberto, Nicola Mozzillo, Francesco M Marincola, Magdalena Thurin

**Affiliations:** 1 Istituto Nazionale Tumori, Fondazione “G. Pascale”, Naples, Italy; 2 Center for Cancer Research, National Cancer Institute, National Institutes of Health, Bethesda, MA, USA; 3 Plastic and Reconstructive Surgery, Regional Melanoma Refferral Center – S.M. Annunziata Hospital, Florence, Italy; 4 Medical Oncology and Immunotherapy, University Hospital of Siena, Istituto Toscano Tumori, Siena, Italy; 5 WHO Melanoma Program, Milan, Italy; 6 Nodality Inc, South San Francisco, USA; 7 Department of Medical Oncology, Fondazione IRCCS Istituto Nazionale dei Tumori, Milan, Italy; 8 Cancer Institute Gustave Roussy, Villejuif, Paris-Sud, France; 9 John Wayne Cancer Institute, Santa Monica, CA, USA; 10 Department of Surgery, Massachusetts General Hospital, Harvard Medical School, Boston, MA, USA; 11 Laboratory of Molecular and Tumor Immunology, Earle A. Chiles Research Institute, Robert W. Franz Cancer Center, Providence Portland Medical Center, Portland, OR, USA; 12 Department of Molecular Microbiology and Immunology, Oregon Health and Science University, Portland, OR, USA; 13 University of Chicago, Chicago, IL, USA; 14 INSERM, U872, Laboratory of Integrative Cancer Immunology, Paris F-75006, France; 15 Université Paris Descartes, Paris, France; 16 Centre de Recherche des Cordeliers, Université Pierre et Marie Curie Paris 6, Paris, France; 17 Tisch Cancer Institute, Icahn School of Medicine at Mount Sinai, New York, NY, USA; 18 1st Department of Medicine, Medical School, University of Athens, Athens, Greece; 19 Center for Melanoma Research and Treatment, California Pacific Medical Center Research Institute, San Francisco, CA, USA; 20 Rush University, Chicago, IL, USA; 21 Royal Marsden NHS Foundation Trust, London, UK; 22 Dermatology/Medicine, UCLA Geffen School of Medicine and Jonsson Comprehensive Cancer Center, Los Angeles, CA, USA; 23 Humanitas Clinical and Research Institute, Rozzano, Italy; 24 Fred Hutchinson Cancer Research Center, Seattle Cancer Care Alliance, University of Washington, Seattle, WA, USA; 25 Leiden University Medical Center and ISA Pharmaceuticals, Leiden, The Netherlands; 26 Peter MacCallum Cancer Centre, East Melbourne, Australia; 27 Unit of Cancer Genetics, Institute of Biomolecular Chemistry, National Research Council, Sassari, Italy; 28 Vanderbilt University Medical Center, Nashville, TN, USA; 29 Tumor Immunology Program, Jonsson Comprehensive Cancer Center (JCCC), David Geffen School of Medicine, University of California Los Angeles (UCLA), Los Angeles, CA, USA; 30 Institute of Medical Immunology, Martin Luther University Halle-Wittenberg, Halle, Germany; 31 Vanderbilt-Ingram Comprehensive Cancer Center, Nashville, TN, USA; 32 Global Early Clinical Development, Clinical Immunotherapeutics, Immunotherapeutics, GlaxoSmithKline Vaccines, Rixensart, Belgium; 33 University of Pittsburgh Cancer Institute, Pittsburgh, PA, USA; 34 Cancer and Inflammation Program, Center for Cancer Research, NCI, NIH, Frederick, MD, USA; 35 Institute of Applied Molecular Medicine (IMMA), CEU-San Pablo University and HM-Hospitals School of Medicine, Boadilla del Monte, 28668, Madrid, Spain; 36 Infectious Disease and Immunogenetics Section (IDIS), Department of Transfusion Medicine, Clinical Center and Center for Human Immunology (CHI), NIH, Bethesda, MD, USA; 37 Sidra Medical and Research Centre, Doha, Qatar; 38 Cancer Diagnosis Program. NCI, NIH, Bethesda, MD, USA

## Abstract

Recent insights into the genetic and somatic aberrations have initiated a new era of rapidly evolving targeted and immune-based treatments for melanoma. After decades of unsuccessful attempts to finding a more effective cure in the treatment of melanoma now we have several drugs active in melanoma. The possibility to use these drugs in combination to improve responses to overcome the resistance, to potentiate the action of immune system with the new immunomodulating antibodies, and identification of biomarkers that can predict the response to a particular therapy represent new concepts and approaches in the clinical management of melanoma. The third “Melanoma Research: “A bridge from Naples to the World” meeting, shortened as “Bridge Melanoma Meeting” took place in Naples, December 2 to 4^th^, 2012. The four topics of discussion at this meeting were: advances in molecular profiling and novel biomarkers, combination therapies, novel concepts toward integrating biomarkers and therapies into contemporary clinical management of patients with melanoma across the entire spectrum of disease stage, and the knowledge gained from the biology of tumor microenvironment across different tumors as a bridge to impact on prognosis and response to therapy in melanoma. This international congress gathered more than 30 international faculty members who in an interactive atmosphere which stimulated discussion and exchange of their experience regarding the most recent advances in research and clinical management of melanoma patients.

## Introduction

**Figure 1:**
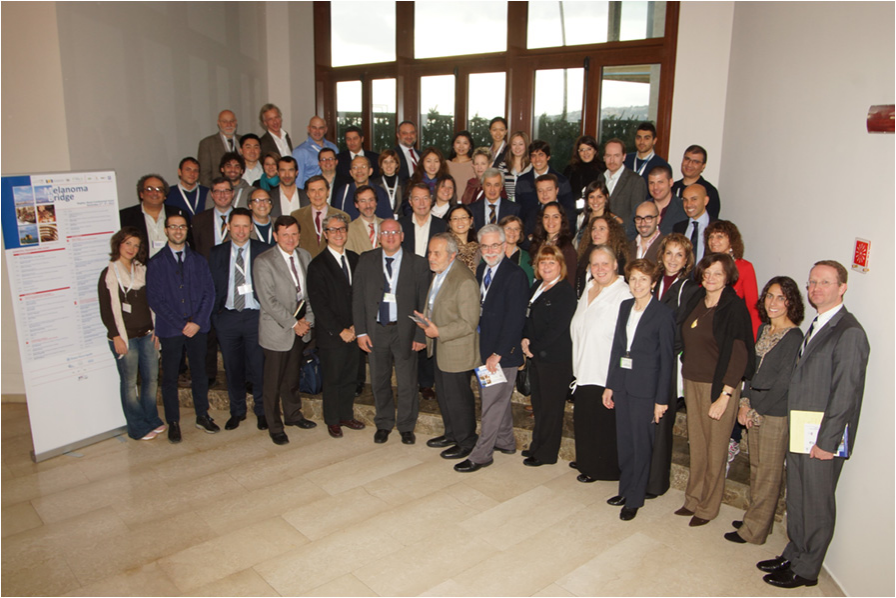
Faculty and some attendants of the Bridge meeting in Naples.

The 3rd “Melanoma Research Bridge” meeting was held in Naples on December 2 to 4th, 2012 (Figure 1). Four topics were mainly discussed during the three-day meeting: molecular advances and biomarkers, combination therapies, novel concepts for integrating biomarkers and novel treatments, and the relevance of biology of tumor microenvironment to treatment of melanoma. In the opening lecture Natale Cascinelli discussed the history of melanoma diagnosis and treatment. Following the consensus conference among clinicians, surgeons, dermatologists, and pathologists in 1967, the histopathologic prognostic factors by Clark (1969) [1] and Breslow (1970) were introduced to determine prognosis and make decisions regarding surgical and adjuvant therapy for patients with cutaneous melanoma. Since then, the prognosis and treatment decisions regarding surgical and adjuvant therapy for a patient with cutaneous melanoma have been based on the current AJCC/UICC (American Joint Committee on Cancer/Union for International Cancer Control) criteria, which include histological and morphologic analysis of the tumor tissue, the anatomic site of origin, and assessment of local spread using TNM staging procedures.

The most recent version of the AJCC (7th Edition) recommended including mitotic rate into the staging system as independent prognostic factor. The change was approved by the UICC. However, histopathological characteristics cannot always accurately predict who will relapse and who will remain disease free. Therefore, additional prognostic and predictive markers to determine the potential for metastatic relapse at the time of diagnosis and to guide therapeutic decisions in adjuvant settings even in early stage melanoma patients are urgently needed. Recently, a new molecular classification of melanoma is evolving based on chromosomal aberrations, gene mutations and signaling pathways activation that underlies biologically distinct subsets of melanoma requiring different clinical management. These approaches have already been proven successful in development of novel molecular diagnostics and importantly novel therapy approaches for melanoma patients.

Melanoma has historically been refractive to chemotherapy which provided very low response rates and little to no benefit in overall survival (OS). The meta-analysis of different Phase II Cooperative Group trials in metastatic Stage IV melanoma showed a median survival time of 6.2 months, 25.5% of the patients alive at 1 year, and a median progression free survival (PFS) of 1.7 months [2]. In recent years, multiple targeted and immune-based therapeutic strategies have been investigated and led to innovative therapeutic approaches in melanoma targeting molecules within activated signaling pathways or the regulatory molecules expressed on the cell surface of activated T cells. The recent approval by the FDA of two drugs for the treatment of metastatic melanoma, including vemurafenib that targets the BRAF harboring V600E codon mutation and the immune response stimulatory monoclonal antibody (MAb) ipilimumab blocking CTLA-4 on T cells can be attributed to an improved understanding of the genetics of the disease and its immune microenvironment, respectively.

Identification of oncogenic mutations in serine/threonine (Ser/Thr) kinase BRAF resulting in valine to glutamine substitution at codon 600 (V600E) in cutaneous melanoma led to development of an effective inhibitors and clinical trials with vemurafenib [3] and other BRAF inhibitor dabrafenib [4]. Vemurafenib is the first BRAF inhibitor developed and approved for the first and second line treatment of metastatic melanoma patients harboring BRAF V600 mutation. Treatment with vemurafenib improved OS, PFS, and response rate (RR), when compared with standard chemotherapy with dacarbazine (DTIC), and showed a typical toxicity profile with photosensitivity reactions, rash, elevated liver enzymes and development of cutaneous squamous cell carcinoma. Response to vemurafenib treatment results in dramatic rates of initial tumor regression (most of them disappear at the PET-CT scan) in about few weeks and rapid but short lasting improvement of symptoms. As demonstrated by phase III trial BRIM-3 treatment with vemurafenib resulted in much better PFS compared to standard chemotherapy (5.3 months in the vemurafenib group versus 1.6 months in dacarbazine group). Overall, the introduction into the clinical practice of vemurafenib as well as of the other recent drug targeting kinases within key signaling pathways (MEK, KIT, alternative BRAF mutations, etc.) identified a critical role to the assessment of the mutational status of these genes in melanoma. Indeed, it is now mandatory to evaluate the BRAF, NRAS and KIT mutational status, to choose the right therapy for the individual patient. The challenge however is availability of well validated and accurate tests that will detect low level and all treatment-sensitive mutations.

Two antibodies that block cytotoxic T lymphocyte-associated antigen 4 (CTLA-4) augmenting antitumor immunity, have been evaluated in phase III clinical trials i.e., ipilimumab (Bristol-Myers Squibb) and tremelimumab (MedImmune, Inc.). Ipilimumab, compared with vaccine therapy or placebo, showed to improve overall survival (OS) of metastatic melanoma patients, with a less impact on responses and PFS. Another promising immunotherapy strategy in melanoma is targeting programmed cell death-1 (PD1) receptor on activated T cells or its ligand PD-L1. Despite improvement in clinical responses with these agents only a subset of patients benefit from CTLA-4 blockade. To better control the response to these drugs new parameters of response, the immune related response criteria, an evolution of the RECIST criteria, and new parameters of management of the immune-related adverse events (irAEs) or adverse events of special interest (AEOSI) are required. Furthermore, biomarkers that can predict response to immunotherapy are much needed tools to help guide treatment decisions for these patients. Thus the emerging era of a personalized approach to the management of melanoma patients will require the identification of the specific subset of melanoma based on the driving mutations or immune response-based biomarkers to design the best drug combination for the individual patient.

## Molecular advances and biomarkers

There is a great need to accurately establish diagnosis, prognosis and to define the outcome of individual melanoma patients but the existing clinicopathologic prognostic factors are not always adequate. In addition, predictive markers to determine the efficacy of treatment at the time of diagnosis and to guide therapeutic decisions for individual patients in adjuvant settings even in early stage melanoma patients emerge as an integral part of clinical management in melanoma. The focus of this section was on emerging prognostic and predictive biomarkers for melanoma as well as novel approaches providing increased opportunities for clinical application of individual markers or multimarker assays including molecular profiling, immune monitoring and functional multiparameteric assay.

Melanoma-associated antigen A3 (MAGE-A3) is a member of MAGE cancer-testis multigene family is melanoma-specific but is not expressed in normal cells. The specific expression of MAGE-A3 on various tumors provides the opportunity for a specific targeted therapy such as vaccines or adoptive T cell therapy and it serves as an eligibility factor for such treatments as only patients with melanoma expressing the antigen (MAGE-A3+) will be selected for treatment. In addition, the specific expression of MAGE-A3 gives the potential for limited off-target effects and no immune tolerance. MAGE-A3 antigen is present in major tumor types, e.g., melanoma up to 76%, multiple myeloma 60%, head and neck cancer 49%, lung cancer 35%, and thus potentially multiple therapeutic indications exists for these tumors including vaccines or T cell adoptive therapy targeting MAGE-A3 [5].

As demonstrated by pre-clinical research the MAGE-A3 protein is weakly immunogenic by itself, but formulation with various immunostimulant is able to induce a stronger immune response leading to improved protection against tumour growth in selected preclinical models [6]. Seventy five patients with unresectable stage III and IV M1a metastatic melanoma were treated with MAGE-A3 vaccine formulated with two different immunostimulants, AS02B and AS15 in a Phase II (GlaxoSmithKline) clinical trial. The AS15 containing formulation showed better immunological response as demonstrated by higher CD4+ count, MAGE-A3 specific antibody titer and clinical activity. The overall survival for the AS02B group of patients was 19.9 months while it was 31.1 months for the AS15 treated group of patients (median follow up of 48 months). Vaccine therapy was well tolerated with mostly grade 1 and 2 toxicities and no noticeable difference in toxicity was observed between AS15 and AS02B groups. No signs of autoimmunity were observed [7].

Gene expression profiling using Affymetrix HG-U133 Plus 2.0 microarray platform was used to identify a signature predictive to the clinical benefit of the MAGE-A3 vaccine treatment using pre-treatment tumor biopsies. A gene signature classifier based on differentially expressed genes was identified. In fact, overall survival in patients stratified by gene signature (GS) positivity was better than overall survival in the non-responder subgroup. Canonical pathway analysis of relevant genes identified immune related signature including genes for antigen presentation, protein ubiquitination and Interferon (IFN) signalling suggesting that the presence of immune effector cells in the tumor microenvironment predicts clinical benefit in response to treatment with MAGE-A3 vaccine [8]. Prospective validation of the signature is under evaluation in a pivotal phase III trial DERMA trial with MAGE-A3 vaccine as an adjuvant treatment for resected IIIB and IIIC melanoma patients (with macroscopic lymph node) (1300 patients planned) and tests whether pre-selection of patients with this GS would allow for enrichment of patients with higher likelihood of response to MAGE-A3 vaccine treatment.

Examples of tumor antigens similar to MAGE-A3 include other Cancer/Testis antigens such as NY-ESO-1 and frequently mutated antigen TP53 that are immunogenic only in cancer patients. Various methods are available to identify immunogenic antigens and rationally design optimally effective cancer vaccines. Serological expression cloning (SEREX) and antibody profiling of protein arrays (Seromics) are tools for the discovery of immunogenic tumor antigens. Spontaneous antibody (Ab) titers against various tumor antigens have been detected in the serum of melanoma patients, non-small cell lung cancer patients and many other cancer types, but not in healthy donor sera can also serve as tumor biomarkers. Correlation of NY-ESO-1 serum antibody with clinical course following anti-CTLA-4 treatment with ipilimumab has been also established [9]. Melanoma patients seropositive at baseline for NY-ESO-1 have a better response rate and outcome than baseline NY-ESO-1 seronegative patients after ipilimumab treatment. After local irradiation and ipilimumab treatment in a melanoma patient, there are changes observed in NY-ESO-1 immunity. Some 40 clinical trials centered on NY-ESO-1 within the Cancer Vaccine Collaborative (CVC) research program established at the Ludwig Institute (New York) in different types of cancer have been completed or are ongoing [10].

Active immunotherapy may present an advantage if able to induce high quality immune responses when they fail to happen spontaneously. It is very important of characterize the mechanisms leading to spontaneous immunity against tumor antigens, and spontaneous immunity can be modulated with immunotherapy (e.g., ipilimumab, and possibly vaccines). The future of immune therapy will combine vaccines that stir the immune system toward the specific tumor antigen with nonspecific modulators of immune suppression such as anti-CTLA-4 or anti-PD1, as well as other drugs that broadly stimulate an array of immune cells including antigen-presenting cells (APCs). One promising group of immunomodulators is the Toll like receptors (TLR) agonists such as Cytosine phosphate Guanine (CpG) oligonucleotides that has strong immunostimulatory properties.

To inform on antigen heterogeneity and local responsive/suppressive environment, it will be very important to measure immune biomarkers in the periphery. These multiple sources of heterogeneity suggest that the therapy should be consistent with testing of tumor phenotype and specific antigen expression in individual patients as well as their immune profile. Novel approaches for detection of circulating tumor cells, circulating DNA, exosomes are explored to detect phenotypic characteristic of tumor cells that express specific combination of surface markers, driver mutations or tumor derived products as well as for immune-response monitoring.

Expression of a subset of chemokine genes is associated with presence of CD8+ T cells in melanoma metastases, so that is possible to identify patients with clinical benefit from vaccines. In fact, the “inflamed” tumor gene expression profile also may be associated with clinical benefit to ipilimumab. Two broad categories of melanoma metastases can be selected, according to gene expression profiling and confirmatory assays identifying a T cell “poor” and a T cell “rich” group. The possibility that a T cell-inflamed tumor microenvironment may be a predictive biomarker for clinical benefit from vaccination is being tested prospectively in the GSK-Bio MAGE-A3 vaccine trials. Innate immune “sensing” of tumors appears to occur via an endoplasmic reticulum-associated molecule referred to as STING (stimulator of IFN genes)-dependent pathway and host type I IFN response. “Inflamed” tumors likely are not rejected due to dominant immune suppressive mechanisms, including indoleamine 2, 3, dioxygenase (IDO), PD-L1, T regulatory cells (Tregs), and T cell-intrinsic anergy. Importantly, all of these are being targeted therapeutically in early phase clinical trials. Combinatorial blockade of selected inhibitory pathways is therapeutically synergistic in preclinical models *in vivo*. Increased PD-L1, IDO, and Tregs in the tumor site are driven by CD8+ T cells in the tumor microenvironment. A new set of surface markers driven by early growth response gene (EGR2) that is a transcriptional target in T cell anergy may provide a strategy for identifying intrinsically dysfunctional CD8+ T cells from tumors, and may also be therapeutic targets.

Three drugs are currently approved for immunotherapy in melanoma: Interferon is the only approved agent for the adjuvant therapy of melanoma, while ipilimumab is approved for metastatic melanoma as first and second line in US and second line in Europe. Interleukin-2 (IL-2) is approved for metastatic melanoma but is not currently in use in Europe. The mechanism by which IFN exerts an antitumor effect has long been debated. The immunomodulatory role of IFN is unclear, but it modulates the immune response, and has anti-proliferative, anti-vascular and pro-apoptotic effects. Type I IFNs (alpha and beta) promote proliferation and clonal expansion of CD4 and CD8 T cells, enhance antibody production of B cells, increase cytotoxic activity of natural killer cells (NK) and CD8 T cells and have negative effects on the activation and proliferation of Tregs.

Attempts to identify patients who benefit from adjuvant treatment with IFN have been undertaken almost from the point of the discovery of IFN’s benefit but to date the results of these efforts have largely been disappointing. The identification of predictive markers that permit selection of patients who are most likely to benefit would allow us to avoid the toxicity of treatment, in more than half of patients who are now offered this therapy. As emerged from meta-analyses, subgroup analyses of randomized trials and translational research studies pre-selection of patients is still controversial. The Wheatley meta-analysis of 2007 [11] show 5-year relapse free survival (RFS) and OS differences of 7% and 3%, while the Mocellin meta-analysis of 2010 [12], analyzing 14 randomized controlled trials from 1990 to 2008 (8122 patients), showed that IFN statistically significantly improved disease free survival (DFS) (Risk Reduction = 18%) and OS (Risk Reduction = 12%) as adjuvant therapy for melanoma patients but no optimal IFN-α dose and/or treatment duration or a subset of patients more responsive to adjuvant therapy was identified.

Most randomized controlled trials evaluating adjuvant therapy with IFN used Breslow thickness and lymph node invasion for staging. Subgroup analyses in randomized controlled trials have been performed by stratification according to clinical and/or pathological features at randomization, not identical over time *(as assessment of risk evolved over time).* Statistical analysis was performed as specified *(intent to treat population), while* subgroup analyses were not pre-specified *(generally not been appropriate as the statistical power of the studies was based on the original overall trial analysis) and* has nonetheless been pursued within and across trials *(determine whether the effects of treatment might be confined to one or another subgroup).* For all of these reasons data emerging from these trials were discordant.

Studies have been performed in tissue and peripheral blood (DNA and serum) of patients receiving adjuvant or neo-adjuvant treatment with IFN to evaluate immune response correlating with clinical outcome. As an example, 20 patients with palpable lymph node metastases (AJCC stage IIIB and C) participated in a neo-adjuvant study [13]. They underwent surgical biopsy at study entry followed by complete lymphadenectomy after induction treatment with high-dose IFN-α (HDI). This study was designed to assess clinical and pathologic responses after 4 weeks of therapy and immunohistochemical evaluation of immune cell subsets and melanoma-associated antigens. Clinical responders had significantly greater increases in endotumoral CD11c^+^ and CD3^+^ cells compared with non responders. No changes have been found in the expression of melanoma-associated lineage antigens, tumor cell proliferation, angiogenesis, or apoptosis were evident.

Additional study in an effort to understand the effects of HDI in relation to the balance of phosphorylated signal transducer and activator of transcription (STAT) pSTAT1 and pSTAT3 have been undertaken. STAT1 and STAT3 were evaluated jointly as mediators of IFN-α effects in the setting of the prospective neo-adjuvant trial [14]. The Janus-activated kinase/STAT pathway of IFN signaling is important for immuneregulation and tumor progression. STAT1 plays a prominent role in the effector immune response, whereas STAT3 is implicated in tumor progression and down-regulation of the response to type I IFNs. Double immunohistochemistry (IHC) for pSTAT1 and pSTAT3 were performed on paired fixed (9 patients) or frozen (12 patients) biopsies. HDI was found to up-regulate pSTAT1, whereas it down-regulates pSTAT3 and total STAT3 levels in both tumor cells and lymphocytes. Higher pSTAT1/pSTAT3 ratios in tumor cells pretreatment were associated with longer overall survival (p = 0.032). The pSTAT1/pSTAT3 ratios were augmented by HDI both in melanoma cells (p = 0.005) and in lymphocytes (p = 0.022). Of the immunologic mediators and markers tested, TAP2 transporter was augmented by HDI (but not TAP1 and MHC class I/II) [14].

Serum multiplexed immunobead-based cytokine profiling can be used to detect melanoma cells and select patients who may be more susceptible to IFN. Powerful high-throughput multiplex immunobead assay technology (xMAP, Luminex Corp.) was used to simultaneously test 29 cytokines, chemokines, angiogenic as well as growth factors, and soluble receptors in 179 patients with high melanoma and 378 healthy individuals, participating in E1694 study. IFN treatment was correlated with decreases in levels of vascular endothelial growth factor (VEGF), epidermal growth factor (EGF) and hepatocyte growth factor (HGF) and increased levels of anti-angiogenic IFN-γ inducible protein 10 (IP-10) and IFN-α. Pretreatment levels of IL-1α, IL-1β, IL-6, TNF-α, and chemokines MIP-1α and MIP-1β were found to be significantly higher in the serum of patients with longer RFS values [15].

Methylthioadenosine phosphorylase (MTAP) a house keeping gene is constitutively expressed in most normal cells and tissues. Loss of MTAP activity was related to deletions in human chromosome 9p21, encoding the tumor suppressor genes CDKN2A and CDKN2B, MTAP and IFN alpha and beta and to epigenetic regulation by promoter hypermethylation. In malignant melanomas, selective deletions in this chromosomal region or promoter hypermethylation are known to result in a loss of MTAP protein expression. MTAP expression has significant impact on STAT1 activity. Using tissue microarrays assembling 465 nevi, primary melanomas and metastases, the expression of MTAP was investigated. In subgroup analysis of patients with tumor thickness of 1.5-4.0 mm revealed a significant survival benefit from adjuvant IFN treatment regarding RFS (p < 0.05) if MTAP expression was observed in the primary melanoma. Patients with STAT1 positive melanomas also tended to benefit from IFN concerning RFS (p = 0.074) and showed a significant benefit of OS (p < 0.05) [16].

Peripheral blood lymphocytes (PBL) from melanoma patients have reduced phosphorylation of STAT1 upon IFN-α stimulation, demonstrating a defect in Type I IFN signaling. Such defects could be partially restored by prolonged stimulation with IFN. Archived peripheral blood mononuclear cells (PBMCs) from 14 Stage IIIB-C melanoma patients pre and post treatment were analyzed for STAT1-Y701 phosphorylation (pSTAT1) levels by phospho-flow cytometry. Significant increase in STAT1 activation in peripheral blood T cells, but not B cells, upon IFN-stimulation was evidenced from Day 0 to Day 29. Moreover this increase of pSTAT1 in peripheral blood T cells also correlated with good clinical outcome. Between patients who showed increased pSTAT1 signaling after HDI therapy, only those who displayed modest augmentation had good outcome.

A multi-factorial genetic model for prognostic assessment of high risk melanoma patients receiving adjuvant IFN has been performed, analyzing data of 284 melanoma patients. In univariate analysis of five-marker genotyping signature was prognostic for melanoma overall survival. This signature defines high and low risk groups and it was shown to be an independent predictor of OS, after controlling for stage [17].

Specific human leukocyte antigen (HLA) class I and II antigens (eg., HLA-A1, HLA-A11, HLA-Cw7, and HLA-DQ1), have previously shown an association with response to interferon therapy or overall survival of patients with metastatic melanoma. A total of 284 high-risk melanoma patients participating in a randomized trial and 246 healthy controls were molecularly typed for HLA class I and II [18]. Specific allele frequencies were compared between the healthy and patient populations, as well as presence or absence of these in relation to recurrence. No allele was associated with absence of recurrence of melanoma in patients receiving adjuvant IFN therapy with the exception of HLA-Cw* 06; -positive melanoma patients who have better relapse-free and overall survival. Alleles related to autoimmune disease were also investigated and HLA-Cw* 06 allele also correlated with psoriasis. In addition, the ulceration of primary tumor and the proinflammatory gene expression profile in tumor can also be considered as a predictive marker of response to adjuvant IFN therapy in melanoma patients. This could be probably due to the activity of IFN in mediating the up-regulation of HLA class I on melanoma cells, and ulcerated melanomas have been demonstrated to present a high MHC class I expression.

In conclusion, the selection of patients for IFN therapy should consider parameters as ulcerated primary melanoma which will be prospectivly validated in EORTC 18081 trial, evaluation of level of biomarkers such as STAT1 ↑, STAT3 ↓, pretreatment levels of proinflammatory cytokines, and patients with HLA Cw*06 polymorphism.

Several polymorphisms have been found within the CTLA-4 gene were shown to have an association with the development of autoimmune disease as Graves’s disease, type 1 diabetes and Addison’s disease. Specific CTLA-4 polymorphisms have previously shown an association with autoimmune symptoms and response to ipilimumab (i.e., GG allele of JO30). GG is associated with decreased expression of CTLA-4 upon T-cell activation and thus a higher proliferation of T-cells. Cohort of 286 melanoma patients treated with high-dose adjuvant IFN in a randomized trial and 288 healthy controls were genotyped for six CTLA-4 polymorphisms previously suggested to be important, AG 49, CT 318, CT 60, JO 27, JO30 and JO 31 [19]. Specific allele frequencies were compared between the healthy and patient populations, as well as presence or absence of these in relation to recurrence. Alleles related to autoimmune disease were also investigated. No significant differences were found between the distributions of CTLA-4 polymorphisms in the melanoma population compared with healthy controls. Relapse free survival (RFS) and OS did not differ significantly between patients with the alleles represented by these polymorphisms. No correlation between autoimmunity and specific alleles was shown.

Ipilimumab, an anti CTLA-4 specific monoclonal antibody therapy, results in a durable clinical benefit for a subset of patients with refractory melanoma and has reversible mechanism-based side effects. Though, definite correlates of clinical response are not established several potential biomarkers of response positively correlate with improved clinical outcome following ipilimumab therapy. The absolute lymphocyte count (ALC) in patients who achieved clinical benefit from ipilimumab had a greater mean increase in ALC after starting therapy than patients who had progressive disease. In addition, to efforts monitoring T cell subpopulations during treatment with CTLA-4 blockade, characterization of antigen specific antibody and T cell responses has similarly led to associations between immunologic changes and benefit from CTLA-4 therapy. Serological studies have evaluated antibody responses against a number of tumor associated antigens, including MAGE, Melan-A, MART-1, gp-100, and Tyrosinase. Patients, who had detectable humoral responses against the cancer-testis antigen, NY-ESO-1 were more likely to experience clinical benefit than those with negative antibody titer.

Gene expression analysis of flow-cytometry purified CD4^+^ and CD8^+^ T cells was employed to assess gene profiling changes induced by ipilimumab. Selected molecules were further investigated by flow cytometry on pre, 3-month and 6-month post-treatment specimens. Ipilimumab up-regulated Ki67 and ICOS on CD4^+^ and CD8^+^ cells at both 3- and 6-month post ipilimumab (p ≤ 0.001), decreased CCR7 and CD25 on CD8^+^ at 3-month post ipilimumab (p ≤ 0.02), and increased transcription factor Gata3 in CD4^+^ and CD8^+^ cells at 6-month post ipilimumab (p ≤ 0.001). Increased EOMES^+^CD8^+^, GranzymeB^+^ EOMES^+^CD8^+^ and decreased Ki67^+^EOMES^+^CD4^+^ T cells at 6 months were significantly associated with relapse (all (p ≤ 0.03). Decreased Ki67^+^CD8^+^ T cells were significantly associated with the development of irAE (p = 0.02). At baseline, low Ki67^+^EOMES^+^CD8^+^ T cells were associated with relapse (p ≤ 0.001), and low Ki67^+^EOMES^+^CD4^+^ T cells were associated with irAE ((p ≤ 0.008). Up-regulation of proliferation and activation signals in CD4^+^ and CD8^+^ T cells were pharmacodynamic markers for ipilimumab. Ki67^+^EOMES^+^CD8^+^ and Ki67^+^EOMES^+^CD4^+^ T cells at baseline merit further testing as biomarkers associated with outcome and irAEs, respectively [20].

A systems-level approach is required for comprehensive understanding of the interconnected components, pathways, and cell types associated with an immune response. Single cell network profiling (SCNP), is a novel platform for assessing and measuring immune function/dysfunction at a “systems” level. SCNP is a multiparametric flow-cytometry based analysis that can simultaneously measure, at the single cell level, both extracellular surface markers and changes in intracellular signaling proteins in response to extracellular modulators [21]. Measuring changes in signaling proteins following the application of an external modulation informs on the functional capacity of the signaling network which cannot be assessed by the measurement of basal signaling alone (e.g., hypo or hyper-responsiveness of a specific pathway). In addition, the simultaneous analysis of multiple pathways in multiple cell subsets can provide insight into the connectivity of both cell signaling networks and immune cell subtypes. The integration of four different parameters makes SCNP technology unique: First, the level of biologic resolution provided, i.e. single cell level. In the SCNP assay, the measurements of post-translational protein changes after exposure to extra-cellular modulators (such as cytokines, chemokines and pharmacologic agents) are made at the single cell level since the technology is flow cytometry based; Second, the type of assessment performed, i.e., cell function. Unlike the “snapshot” view of cellular signaling provided by measuring the basal or resting phosphorylation state of an intracellular protein, the application of an extra-cellular modulation forces the intracellular signaling network to respond, revealing dynamic information about the way the network processes information. Thus, the functional capabilities of key signaling networks can be compared, for instance, between the cells of healthy individuals and diseased patients, allowing detection and characterization of signaling abnormalities associated with disease or in the same patient over time (e.g., disease monitoring), allowing the identification of changes associated with disease progression or with response to therapeutic agents or between patients, allowing for patient stratification. Third, the type of measurement performed, i.e., quantitative and multiplexed. Since different modulators can act on the same intracellular pathways and, in heterogeneous tissues, on multiple cell subsets at the same time, the SCNP approach allows measurement in a quantitative fashion and simultaneously of changes in intracellular protein levels in response to different modulators in different cell subpopulations without the need for cell separating/sorting. Finally but importantly, for such an approach to be useful not only in the research context but also in a clinical one (e.g., development of clinical actionable biomarkers for disease status or response to treatment), it must be highly repeatable and reproducible, meeting the regulatory standards of analytic validity [22]. This has been recently achieved with coefficients of variations (CV) of functional assays pathway stimulation routinely below 5%. Achieving this goal requires strict instrument standardization and performance monitoring, rigorous attention to sample quality and reagent qualification, and the introduction of automation and validated methodology and ad hoc informatics infrastructure [22].

When applied to pathways shown to be important in disease pathology, this method of mapping signaling networks has potential applications in the development of disease profiling, the identification of novel disease targets, predictive tests for therapeutic response and patient selection and overall for improved efficiency of drug development [23,24]. Recently, using this technology have published a “map” of the healthy immune signaling network, in which several age-associated signaling nodes were identified in specific subsets of cells within PBMC samples from healthy individuals [25]. These studies underscore the potential utility of SCNP for immune monitoring applications, as well as biomarker development for immune-mediated diseases. To this regard, a study which has direct relevance to the ipilimumab mechanism of action since it examined CD4+ T cell signaling in the context of CTLA-4 expression was performed [26]. Results showed that signal transduction activities differed between CTLA-4 defined CD4+ subsets, and between healthy and melanoma samples. Further studies are ongoing, which will expand on the biological findings and assess the association between ipilimumab response and signaling differences. Taken all together these studies demonstrate the utility of SCNP for immune monitoring and point to the promise of using this method for cancer immunotherapy biomarker development.

Better methods to determine melanoma progression would allow further improvements in the prognostic assessment of melanoma patients. Importantly, the accurate prognosis will benefit proper risk stratification of the early stage melanoma patients for adjuvant treatment. The diagnosis of primary cutaneous melanoma at the pathological level can represent a daunting task. Several features are used to diagnose melanoma, such as cytologic atypia, maturation with descent, poor circumscription, presence of mitoses, and asymmetry. However, the pathologic diagnosis of melanoma remains challenging, resulting in a high degree of inter-observer variability Although traditional cancer diagnostics approaches including histopathology and IHC will likely remain standard tools for the future the upcoming molecular analyses might be incorporated in the context of these established methods when the definite diagnosis cannot be reached. They potentially might be systematically incorporated and lead to improvement of the AJCC/IUCC system.

Different approaches are routinely explored for discovery and validation of biomarkers to improve diagnosis, classification and prognosis. Gene expression profiling of a series of freshly acquired nevi, primary, and metastatic melanomas identified differentially expressed gene sets during melanoma progression, including nevus to primary melanoma, radial versus vertical growth phase melanoma or primary *vs.* metastatic melanoma. These differentially expressed genes were hypothesized to have potential utility as novel melanoma biomarkers. Immunohistochemical analyses confirmed the differential expression of some of these markers.

A three-marker IHC-based prognostic assay in primary cutaneous melanoma developed and tested in a cohort of 395 patients. The markers evaluated included NCOA3 (nuclear receptor coactivator 3), RGS1 (regulator of G protein signaling), and SPP1 (osteopontin), and were scored both by a pathologist blinded to patient outcomes on a 0–3 scale, and using a digital imaging analysis. The outcome parameters evaluated were disease-specific survival (DSS, primary endpoint) & sentinel lymph node (SLN) metastasis. Each marker alone had been shown to significantly predict DSS and SLN status. Marker expression was then validated in an independent cohort of 141 cases from German Cancer Registry (Heidelberg/Kiel cohort). The multi-marker score was independently predictive of DSS in cohorts, surpassing tumor thickness and ulceration. Ulceration, an established prognostification factor for localized primary melanoma that has been included to the AJCC synoptic for microstaging of melanoma. Thus, the multi-marker assay can be considered a very important prognostic factor to predict DSS and SLN metastasis [27].

More recent studies have focused on the role of the pleckstrin homology domain-interacting protein (PHIP) gene in melanoma progression. PHIP emerged as the gene most highly expressed in melanoma metastases *vs.* primary tumors. PHIP was initially identified through its interaction with the pleckstrin homology domain of insulin receptor substrate (IRS) proteins. While it has demonstrated roles in Insulin-like Growth Factor (IGF) signaling and glucose metabolism, a role in cancer had not been previously reported [28]. Aberrant activation of the IGF signaling Pathway has been demonstrated in different cancers, and for this reason it is a rational target for anti-cancer therapy. IGF binding results in receptor phosphorylation, creating binding sites for IRS docking proteins. IRS activation subsequently recruits PI3K and activates AKT, which has been shown to activate aerobic glycolysis in tumor cells. Glycolytic pathway genes in turn have been shown to be capable of promoting tumor invasion and metastasis.

Based on recent analyses, PHIP can be considered a novel marker and mediator of melanoma metastasis. shRNA-mediated targeting of PHIP significantly reduced murine and human melanoma invasion and metastasis. PHIP mediated its pro-invasive effects by virtue of activating AKT. The prognostic role for PHIP in human melanoma was demonstrated in a study analyzing a cohort of 345 cases. There was a significant association between PHIP levels and ulceration.

Genotypic analysis aimed to identify the molecular subtypes of melanoma demonstrating activated PHIP, based in part on its activation of AKT. AKT activation in melanoma is associated with NRAS mutation or BRAF mutation and PTEN loss. It was hypothesized that if PHIP expression is sufficient for AKT activation, then high PHIP-expressing melanomas should not have mutant NRAS or BRAF/PTEN mutants. The great majority of high PHIP-expressing melanomas were devoid of NRAS or double BRAF/PTEN mutants. Thus, PHIP levels are enriched in triple negative (WT BRAF, NRAS, and PTEN) and double negative melanoma (mutant BRAF and WT NRAS and PTEN).

The PHIP gene normally resides on the 6q14 locus, but in melanoma 6q loss has been reported. By fluorescent in situ hybridization (FISH) analysis, not only is the PHIP locus preserved in melanomas, approximately 80% of high PHIP-expressing melanomas had an increased copy number. More recent analyses were presented indicating a prognostic role for high PHIP copy number. Data were presented from experimental models of melanoma showing that suppression of PHIP results in reduced glycolysis, lower VEGF levels and decreased tumor angiogenesis. On the basis of these results, a model of ulceration development was presented in melanoma in which PHIP activation results in activation of the IGF1R pathway and AKT, ultimately resulting in increased glycolysis, invasiveness, and angiogenesis, resulting in increased capacity to develop both ulceration and metastasis.

Between the mechanisms of acquired (late) BRAF inhibitors (BRAFi) resistance, there is a predominance of MAPK-reactivation. This can happen by RAS mutations, MEK mutations, BRAF V600E/K splicing or amplification. This phenomenon reflects the fact that effective initial on-target MAPK pathway inhibition in the majority of melanomas provides the selective pressure for the tumors to re-activate a melanoma-addicted pathway. By whole-exome sequencing, AKT gain of function mutations were identified in a minority of melanoma with acquired BRAF inhibitor resistance. These AKT mutants conferred BRAF inhibitor resistance most potently in a cells which only weakly upregulated AKT in response to BRAF inhibition. Thus, AKT mutants amplify an adaptive response to BRAF inhibition. This adaptive AKT rebound in response to BRAFi is widespread early during therapy. The landscape of acquired resistance is quite heterogeneous but core pathways exist. In a single patient, both MAPK- and AKT-dependent mechanisms of acquired resistance can concur.

## Combination therapies

Combination therapies are the most promising approach to add the therapeutic value to therapy with a single drug. A major challenge for the combination treatment in melanoma is to overcome intrinsic and acquired resistance. The elucidation of signaling pathways underlying resistance is important in order to develop effective personalized targeted strategies for individual patients’ as multiple mechanisms or resistance develop during the initial treatment. Also, microenvironmental factors and immune response need to be considered in designing appropriate treatment strategies and developing markers predictive of response. In addition, the efficacy of the treatment could depend on using sequential of combined modalities. Finally, preclinical models and methods concerning the profiling of individual tumors need further improvement. Despite, these numerous challenges several combination therapies for melanoma entered clinical evaluation, as discussed during this session.

The prognosis of melanoma varies widely by stage and for high risk surgically resected melanoma (AJCC stage IIB and higher) the risk of recurrence and death exceeds 35-40% at 5 years. On the other hand, most of cases are diagnosed at early stage and in this setting patients with high risk of developing metastatic disease may benefit from adjuvant therapy which presents the best opportunity at curing melanoma at this time. Advanced melanoma displays immunological tolerance and it is hypothesized that the role of immunotherapy in the adjuvant setting could be the greatest before immune tolerance is established. Multiple clinical trials have been performed with adjuvant IFN-α immunotherapy for resected melanoma, and 3 meta-analyses reviewing these trials have been reported to date. The Mocellin meta-analysis of 14 trials reported 18% reduction in the risk of relapse and 12% reduction in the risk of death with the use of adjuvant IFN overall [12].

While most IFN adjuvant trials in melanoma have reported a RFS benefit, a significant impact on OS has been reported only in the high dose IFN regimen as tested in the E1684 trial. E1684 randomized high risk melanoma patients after surgery to either observation or treatment with HDI given intravenously, 5 days a week for 4 weeks (induction) then subcutaneously at lower dosage, 3 days a week, for 48 weeks (maintenance). E1694 randomized patients to be treated with adjuvant ganglioside GM2-KLH/QS-21 (GMK) vaccine for 96 weeks versus HDI. HDI demonstrated significant improvements on RFS and OS in the E1684 (versus observation) and E1694 (versus GMK). In E1684, at the median follow up of 6.6 years, compared to observation, there was a 33% reduction in the hazard of death in favor of IFN 39% reduction in the hazard of recurrence and the greater benefit was seen in patients with a high tumor burden (N1-2b). In E1694, compared to the GMK vaccine, there was 24% reduction in the hazard of mortality and 25% reduction in the hazard of relapse, with the greatest benefit seen in patients with a lower tumor burden (T4, N-) [29].

The EORTC 18991 trial hypothesized that prolonged treatment with IFNα-2b is needed to obtain a maximal anti-angiogenic effect, and thus has compared observation with an intended 5 years of maximally tolerable doses of pegylated IFNα-2b (pFNα-2b) for patients with resected, stage III melanoma (TxN1–2 M0). During the first 8 weeks, pegylated IFNα-2b was administered at 6 mg/kg per week followed by 3 mg/kg per week maintenance therapy for up to 5 years. The study was designed to measure changes in distant-metastasis-free survival (DMFS) as the primary endpoint, and was powered to detect a 9.75% absolute difference in DMFS at 4 years. The study results were first reported in 2007 at a median follow up of 3.8 years and later updated in 2011 at a median follow up of 7.6 years [30]. Significant RFS benefit from pIFNα-2b was consistently seen in the study overall (HR = 0.82 in 2007 and 0.87 in 2011), while there was no overall benefit seen in DMFS or OS. Stratifying patients by stage (N1 versus N2), patients with microscopic nodal involvement (N1) derived significant benefits in RFS and DMFS as reported in 2007; these improvements were still seen in the updated report in 2011, but no longer statistically significant. Patients with clinically detectable nodal involvement (N2) showed no significant benefit in any endpoint. On the other hand, further subgroup analysis showed that the greatest benefit of pIFNα-2b was seen in the N1 subset with an ulcerated primary with a median OS of > 9 years versus 3.7 years. The median duration for the induction phase was 8 weeks, while the median maintenance duration was 14.9 months. In addition, 31% patients discontinued treatment owing to adverse events and 23% remained on treatment in years 4–5; this proportion of treatment attrition due to toxicity is higher than the 10% reported in E1694 [29].

Predictive markers of therapeutic benefit are needed to accelerate progress in the adjuvant therapy of melanoma to treat only those who would relapse and to treat only those who have the capacity to respond. Biomarkers could serve to select patients who would benefit from treatment with IFN and to spare side effects of the treatment for patients who are not likely to respond. Biomarkers of risk & benefit that have been evaluated in studies of adjuvant IFN include S100B, autoimmunity, the proinflammatory cytokine profile in serum. Autoimmunity was identified to be a predictive factor for response in adjuvant treatment with IFN as demonstrated by the presence of thyroid, anti-cardiolipins, anti-nuclear, and anti-DNA autoantibodies [31]. The HLA genotype was also found to be a factor predicting recurrence in patients treated with IFN as an adjuvant. The rate of relapse is significantly lower in patients with HLA A33, B57, Cw03 and Cw06. Other immunomodulatory mechanisms of high-dose IFN are the increase of tumor infiltrating cells, the decrease of circulating Treg cells, the modulation of the STAT1/STAT3 balance in both tumor cells and host lymphocytes, the change in serum cytokine concentrations, and the normalization of T cell signaling defects in the peripheral blood lymphocytes [32]. Candidate biomarkers linked to the pro-inflammatory immune response and markers of immunosuppression as assessed in the tumor microenvironment (TME) and in circulation may have therapeutic predictive roles in relation to immunotherapy. E1609 allows the essential linkage of these biomarkers between the TME and the circulation supported by the common systems biology where we hypothesize that a baseline pro-inflammatory biomarker model/signature will be predictive of therapeutic benefit.

Recently, it has been postulated that the effects of IFN could be even related to the BRAF mutational status in melanoma (Soldano Ferrone, unpublished). BRAF V600E mutation-activated pathway is present in approximately half of cutaneous melanomas. Although, BRAF inhibitors induce rapid anti-tumor responses in about 50% of patients many challenges still exist to optimize BRAFi-based therapy. First, about 40% of patients do not respond to BRAFi due to primary resistance. Second, only about 5% of patients have complete responses. Third, the median duration of response among responders is less than 7 months (secondary resistance). These clinical findings emphasize the need to design rational combinatorial therapeutic strategies to increase the frequency of complete responses and to improve the duration of response to BRAFi therapy. In this regard we are testing the hypothesis that the BRAFi and type I IFN-α combination is more effective than the individual agents in suppressing human melanoma cell growth both *in vitro* and *in vivo*.

The interaction of type I IFN with target cells is mediated through a heterodimeric receptor composed of two subunits referred to as IFNAR1 and IFNAR2 with the binding of this receptor playing a key role in the antiproliferative activity of type I IFN. The available evidence in the literature argues for an IFNAR1 down regulation in melanoma cells harboring a mutated active BRAF [33]. This finding reflects an increased ubiquitination and degradation of IFNAR1 by βTrcp2/HOS protein, an E3 ubiquitin ligase, which is induced by the BRAF-MAPK signaling activity. This mechanism explains why inhibition of the BRAF-MAPK signaling pathway by vemurafenib upregulates IFNAR1 expression in melanoma cells with a mutated active BRAF. Consequently, the combination of IFN and BRAF inhibitor is much more effective as compared to the two single agents in monotherapy, in cell lines both sensitive and resistant to BRAF inhibitor. Moreover, the *in vivo* combination of IFN-α and BRAF inhibitor obtained a significant survival prolongation of SCID mice grafted with BRAF mutated cells, strongly suggesting that metastatic BRAF-mutated melanoma patients could benefit from the combination of IFN and BRAF inhibitor treatment (Soldano Ferrone, unpublished). Clinical trials are being implemented to test whether the therapeutic efficacy of the BRAF-I and INF-α combination is significantly higher than that of the individual agents in patients with melanoma with BRAF-mutation.

Another effect of this combination is the upregulation of HLA Class I molecules as well as of antigen processing machinery (APM) for melanoma antigens, inducing a greater sensitivity to T cell recognition which supports the rationale for the association with other immunotherapies such as anti-CTLA-4 treatment. In a Phase II Trial, tremelimumab was tested in combination with high-dose IFN-α yielded a high overall response rate. Overall, 37 patients were treated with tremelimumab given at 15 mg/kg every 90 days, and high-dose IFN-α simultaneously given at standard FDA-approved doses [34]. Among 35 evaluable patients, this therapy resulted in 4 complete response (CR) and 5 partial response (PR) (response rate, 24%), and 14 (38%) stable disease (SD); median PFS was 6.4 months and median OS 21 months. A rationale for a possible additional combination therapy is based on the blockade of PD1-PDL1 interactions with anti-PD1 or anti-PD-L1 mAbs, which enhance the cytotoxic function of HLA class I antigen restricted, tumor antigen-specific T cells *in vitro* as well as with antitumor effectors in the tumor microenvironment *in vivo*[35]. Since IFN induces tumor B7-H1 expression this provides the basis for the combination of IFN and anti-PD1 treatments. Concluding, IFN might have a role in rational combinations with the new agents designed in a more or logical sequence of drug administrations.

Various studies of combined treatment of chemotherapy and cytokines have been conducted with contradictory results. The phase II E4697 adjuvant trial for high risk resected stage III-IV melanoma tested the hypothesis that granulocyte macrophage colony-stimulating factor (GM-CSF) and/or Tyrosinase peptide-based vaccine would be of therapeutic benefit. However, the trial showed that neither GM-CSF nor peptide vaccination achieved the objectives of significant improvement in OS or DFS. A suggestion of a favorable effect of GM-CSF on DFS and OS among Stage IV subjects was seen at subset analysis that warrants further investigation [36]. Recently, the study of Intergroup S0008 phase III clinical trial has compared HDI versus Biochemotherapy (BCT) [cisplatin, vinblastine, DTIC plus IL-2 and IFN] as adjuvant treatment for patients with high risk melanoma. RFS at 5 years was 47% for BCT and 39% for HDI, median RFS was 4.31 *vs.* 1.9 years, respectively, and OS at 5 years 56% for both [37]. Significant differences in RFS for biochemotherapy without differences in OS suggest that BCT is a valid alternative for adjuvant treatment in patients with high risk melanoma.

Other adjuvant modalities evaluated were cellular vaccines: Canvaxin (was detrimental in Ph III trials), MAGE-A3 peptide vaccination given in adjuvant with a TLR-9 agonist (results are pending), and anti-CTLA-4 blocking monoclonal antibody therapy with ipilimumab (EORTC 18071 and US Intergroup E1609 trials are still pending). Adjuvant trials of BRAFi and BRAFi plus MEKi are ongoing. EORTC 18071 trial randomized patients with resected stage IIIA, IIIB, IIIC melanoma, to receive adjuvant ipilimumab at 10 mg/kg or placebo. Intergroup E1609 Adjuvant Phase III trial randomizes patients with resected stage IIIB, IIIC, M1a or M1b to be treated with ipilimumab at 10 mg/kg or 3 mg/kg versus HDI.

The induction of an effective T cell mediated antitumor response is dependent upon the encounter of cytotoxic T-cells with their cognate antigen. However, the identification of tumor antigens that can successfully mediate an effective antitumor response with ACT immunotherapy requires more research. In mice, the adoptive transfer of T cells specific for a nonmutated melanoma differentiation antigen (gp100) can mediate tumor regression of established B16 murine melanoma [38]. However, B16 does not harbor a BRAF mutation and therefore does not model the human melanoma mutational landscape [39]; consequently, established B16 tumors on C57BL/6 mice are nonresponsive to vemurafenib therapy. Despite its appropriateness as an antigenic system to evaluate “self-reactivity” with gp100 T cell receptor (TCR)-specific transgenic T cells, the efficacy of this sort of T-cell mediated antitumor immunity may not translate well into human clinical trials given the lack of B16 human genetic relevance. Therefore, the development of relevant BRAF V600E mutant murine melanoma transplantable models that allow for high-throughput antitumor efficacy testing in rodents could serve to narrow the existing “bench to bedside” translational gap. The advent of modern genome-wide screening technologies has allowed more comprehensive understanding of the genetic complexity of human melanoma [39,40]. Common co-occurrence of BRAF mutations with deletions of the PTEN tumor suppressor gene [39] suggests a concerted biological contribution to melanoma development. Consistent with this observation, transgenic mice engineered to express the BRAF V600E oncoprotein on a PTEN deficient background develop metastatic melanoma [41]. Established tumors from this syngeneic BRAF V600E/PTEN^−/−^ transplantable melanoma cell line are highly sensitive to orally administered vemurafenib therapy on C57BL/6 immunocompetent hosts. The lack of gp100 expression recapitulates the common intratumoral gp100 expression heterogeneity observed in majority of patients with metastatic melanoma. Efficacy testing of vemurafenib in combination with ACT immunotherapy using this relevant model, did not improve the antitumor activity of adoptively transferred gp100 TCR-specific T-cells. Given the additional heterogeneity observed for other “self” melanoma differentiation antigens (MART-1, Tyrosinase) in human melanoma, preclinical modeling of “non-self” antigen reactivity becomes essential for the evaluation of combinatorial immunotherapeutic modalities. In addition, the development of antigenic systems that target the product of mutated genes may serve as definite proof that neoantigens can mediate successful T-cell antitumor responses against melanoma. Identification of a tumor’s expressed mutated genes by whole-exome sequencing, may allow prediction of mutant T cell epitopes that could be exploited to develop new preclinical models of “non-self” antigen reactivity. Finally, appropriate preclinical modeling of both *in vivo* sensitivity to targeted therapy and antigen-specific immunity (“self” and “non-self”), should better predict the clinical efficacy of therapeutic regimens that combine targeted therapy (e.g. vemurafenib) with a T-cell based immunotherapeutic approach (e.g., ACT immunotherapy).

Adoptive cell transfer (ACT) immunotherapy proves the concept of effective antitumor T cell mediated cytotoxicity and is the most effective form of immunotherapy for patients with metastatic melanoma. Current ACT clinical protocols using autologous TILs can mediate durable complete responses in 22% of patients with 95% of patients surviving beyond 3 years [42]. Therefore, combining two state-of-art-treatments i.e., vemurafenib with adoptive cell transfer immunotherapy in patients with advanced melanoma could prove more effective. It has been reported that BRAF V600E inhibitor improves recognition of human melanoma cells by antigen-specific T lymphocytes through increased expression of melanoma differentiation antigens (MDAs) [43]. In addition, improved tumor recognition is associated with enhanced expression of MHC class I and biopsies of melanomas treated with vemurafenib increases CD8+ T cell infiltrating the tumors. Preclinical studies have also shown that the combination of agents that activate T-lymphocytes such as agonistic antibodies to CD-137 in models of melanoma synergize with BRAF-inhibitors to induce tumor regressions. Blocking activity of oncogenic BRAF can also increase the expression of pro-apoptotic Bcl-2 family members Bim and Bad potentially lowering the intrinsic apoptotic threshold of the tumor cells [44,45]. Furthermore, BRAF inhibition can decrease the production of immune-suppressive cytokines by tumor cells that might otherwise recruit regulatory Tregs and Myeloid-derived suppressor cells (MDSCs) that could hinder a T cell mediated antitumor response [46]. This provides a strong rationale to combine anti BRAF therapy with immunotherapy strategies.

BRAF mutations have been identified as the most frequent in melanoma and development of BRAF inhibitors for clinical setting has been extremely successful. Vemurafenib is a “selective” BRAF Inhibitor of V600 mutant BRAF kinase in melanoma, with an optimal activity at the dose of 960 mg twice daily (bid), as defined by the phase I, II and III clinical trials. Based on the Phase III trial comparing vemurafenib *vs.* DTIC 2011 vemurafenib was approved by the FDA [47]. Concomitantly with the drug, companion diagnostic test that can identify melanoma patients with BRAF V600E mutation who most likely to benefit from this therapy was also approved.

Despite the impressive single-agent clinical activity several problems exist that need to be overcame to improve the efficacy of vemurafenib i.e., toxicity of the drug, reactivation of the MAPK pathway, development of resistance and occurrence of the secondary tumors. Combination regimens based on mechanisms of resistance and/or activation of oncogenic pathways that involve other therapeutic targeting agents for melanoma (MEK, PI3K, AKT, CDK4/6, Hsp90 etc.), are expected to have additive or synergistic effects on clinical outcome when added to BRAF inhibitors. Other promising avenues of melanoma drug development include targeted to the host i.e., immunotherapy (ipilimumab, anti-PDL1, pIFN). Another, possibility is anti-angiogenic therapy (e.g., bevacizumab and IL-2) in combination with targeted therapy (BRAF, MEK, ERK etc.) targeting the tumor.

Other tumors harboring BRAF mutations have also been identified. For example, 10% of colon cancer (CRC) population harboring BRAF mutation has extremely poor prognosis. In the Phase I trial CRC expansion cohort, activity of a single agent vemurafenib has been disappointing (1 PR out of 20 total) [48], but recent preclinical studies suggest, that combination of BRAF plus EGFR inhibitors show strong synergy and may be more effective. The strong synergistic effect between inhibition of BRAF and EGFR is explained by a powerful feedback activation of EGFR caused by BRAF inhibition [49]. These data explain poor clinical response of BRAF V600E colon cancer to vemurafenib monotherapy and provide a strong rationale for a clinical trial combining vemurafenib plus cetuximab in CRC BRAF mutated patients. Vemurafenib has been effective in thyroid cancer with V600 BRAF mutation (2 PR, 1 SD out of 3 total on Phase I trial) and there is already a registration trial ongoing in papillary tumors [50]. Other tumor types have also been shown to carry variable BRAF mutation rates (LCH 60%, HCL 100%, NSCLC 2%, cholangiocarcinoma, breast, ovarian, germ cell etc.). Currently, there is an open label Phase II trial designed to treat all BRAF-mutated tumors (“BRAFomas”) [51] with the next step being to take the most promising leads from this trial to registration or alternatively use this as a blueprint for development of combination strategies.

However, it is virtually impossible to explore all potential drug combinations using tumor material before and after tumor progression or performing sequential biopsies from the patients considering functional complexity of the tumor and developing resistance. Model systems to identify novel combination treatment for cancer are lacking. Possible solutions are: to develop *in vitro* models of resistance, develop tumor sampling techniques (short term cultures, circulating tumor cells/DNA), as well as use mathematical models based on information from patient tumors both at the time of diagnosis and at the time of ultimate resistance to predict more efficacious combinations of existing drugs upfront. *In vitro* models of resistance should allow for observing dynamic changes in melanoma cells in the presence of drugs or drug combinations and provide a better view of drug response than static IC50 single end-point assays. In melanoma cell lines, the response to vemurafenib is different within seemingly isogenic cell lines as some cells respond by entering quiescence, very few cells undergo apoptosis (except in SK-MEL-5) and different subpopulations of cells respond by varying levels of inhibition of proliferation rate [52]. The net result of the response to vemurafenib is that after treatment the entire population of tumor cells begins to rebind, thus mimicking the resistant tumors developing in patients. Another way to overcome limitations of serial tumor biopsies is to obtain cells from patients for *in vitro* assays based on circulating tumor cells (CTCs) or circulating macromolecules that originate in tumor cells. If the DNA/RNA corresponds to the changes observed in individual tumors with sufficient accuracy, they should facilitate detection of the molecular mechanism underlying resistance in individual patients. Novel models mimicking the interaction of both tumor and stromal populations, including TILs are needed. For example, *ex-vivo* cultures that capture these interactions could provide a system closer to individual tumor to optimally guide new treatments and to predict the efficacy of combination of new drugs for metastatic melanoma.

As with all tumors, clinical trials for melanoma are not consistently conducted with a solid rationale from preclinical animal efficacy studies. All preclinical models have limitations and often fail to predict antitumor efficacy in human clinical trials. Mouse models either xenotransplanted or genetically-engineered completely mimicking human melanoma biology are not available and the use of immunodeficient hosts that do not allow to address the effect of the immune system account for the existing translational “bench to bedside” gap. New models are required to develop predictors of response and to establish combination molecular or/and immune-based therapies since some combinations may be antagonistic or affect tumor infiltrating lymphocytes (TIL) effector function.

Maximum BRAF V600E level reduction occurs during Cycle 2 and 3 in vemurafenib treated patients as measured using RT-PCR based assay [53]. The same level of reduction is observed later, in at least Cycle 4 in patients treated with the combination of dabrafenib (BRAF inhibitor) and trametinib (MEK inhibitor) suggesting better response to RAF + MEK combination in comparison to BRAF alone [53]. The reduction of circulating mutated BRAF level is also associated with tumor regression by RECIST. In addition, a detectable increase in BRAF V600E level was seen ~50 days prior to radiographic progressive disease in patients treated with BRAF-directed therapy, thus potentially enabling an early intervention to delay resistance [53]. If the mechanism of resistance can be identified before treatment or early in the treatment the strategies can be developed to use at the beginning of therapy.

The lessons learned from BRAF inhibitor development lead us to conclusion that targeting mutated BRAF gene in melanoma is a valid strategy, but not sufficient to improve overall survival. Careful biochemical and genetic analysis of the tumor samples and/or short term cultures derived from patient’s tumors including stroma and TILs may point to the useful combination treatments for individual patients. Remaining challenges include proper PK/PD investigations as part of Phase I studies of new drug candidates to truly evaluate their therapeutic potential. Importantly, correlative studies should be incorporated in the early drug development including exploration of novel targets, developing resistance, combinatorial strategy testing, and dosing strategies. Computer simulation studies of non-standard drug scheduling may also help to move beyond simple sequential or combination therapy to achieve the goal of improving patients’ outcome.

BRAF inhibitors have different effects in BRAF V600E mutant melanoma and BRAF wild type cells. Reduction of MAPK signaling and consequent block of the cell proliferation in the V600 mutated melanoma cells whereas activating MAPK signaling and progression through the cycle cycle in cells harboring wild type BRAF is observed. The evidence suggests that the use of BRAF inhibitor alone leads to reactivation of the MAPK pathway and enhances its activation through another member of RAF family of kinases CRAF. This “paradoxical” MAPK activation by BRAF inhibitors can result in the development of cutaneous squamous cell carcinoma of keratoacanthoma type (cuSCC/KAs), a typical cutaneous manifestation of BRAF inhibitor therapy such as with vemurafenib or dabrafenib in ~25% of patients [54]. This suggested that combination of BRAF with MEK inhibitors might be preferred option as it might overcome CRAF stimulation, prolong PFS and prevent the emergence of squamous-cell carcinomas. Indeed, this combination in BRIM 7 trial (GDC-0973/cobimetinib and vemurafenib) resulted in preventing the emergence of hyperproliferative skin lesions [55].

In preclinical mouse-models, cyclooxygenase 2 (COX2) inhibitors prevent the formation of squamous cell carcinomas of the skin even in COX-2 deficient mice exposed to DMBA and TPA, a commonly used skin carcinogenesis mouse model. A hypothesis based on these results is that the hyperactivation of COX-2 results in increased prostaglandin E2 (PGE2) level that activates PI3K and Src via G protein coupled receptors EP2/4. PI3K and Src activate HRAS and CRAF, and trans-activate EGFR that results in the paradoxical activation of MEK, during the treatment with vemurafenib. This was demonstrated in another murine preclinical model where COX-2 inhibition with COX-2 inhibitor (celecoxib) blocked acceleration of DMBA/TPA-induced skin tumor development by a BRAF inhibitor [56]. Another analysis of these murine models showed that the addition of celecoxib decreased pMEK and pERK levels. Further studies in a murine skin squamous cell carcinoma cell line, PDV, showed that vemurafenib-stimulated paradoxical growth of PDV cells is inhibited by celecoxib.

As already discussed, vemurafenib was the first BRAF inhibitor that show significant clinical benefit in melanoma patients. Treatment with vemurafenib leads to a reduction in the level of phosphorylated MEK and ERK in tumors containing mutated BRAF V600E which is associated with clinical response. BRAF inhibitors can effectively inhibit 90-100% of MAPK signaling. This underlies the rapid response and clinical effect of this class of drugs on patients with BRAF V600 mutant melanoma. Importantly, it appears that the great majority of patients treated with single BRAF inhibitor will eventually have progressive disease despite successful inhibition of the V600E BRAF and high objective response early in the course of therapy. ERK (and MEK) phosphorylation, Cyclin D1, and Ki-67 are again upregulated in samples taken at the time of progressive disease as compared to their reduced expression early in therapy.

Available data suggest that the resistance to vemurafenib is not related to the development of secondary “gate-keeper” mutations of BRAF V600E gene that prevents the binding of the treatment drug to BRAF. In fact, a large number of resistant melanoma samples have had entire BRAF gene sequenced and no secondary mutation were found. Instead the resistance is mediated by reactivation of MAPK signalling in most tumors through alternative mechanism. Several mechanisms of acquired resistance to BRAF inhibition have been identified including: acquisition of NRAS and MEK1 activating mutations, upregulation of receptor tyrosine kinases (i.e., PDGFRB, ERBB2), PTEN loss, activation of the Ser/Thr MAPK kinases (COT), NF1 loss, BRAF truncations, BRAF amplification and possibly others. All of these mechanisms involve the reactivation of the MAPK pathway [57]. In addition, signalling through the PI3K pathway involving PI3K and AKT through the IGF1R signalling represents an alternative mechanism of acquired resistance. Studying resistance to BRAF inhibitors has also shown that HGF levels can be increased in primary and acquired resistance. Results from the Sequenom Oncocarta Panels, performed prior to therapy with BRAF inhibitors, NRAS, and BRAF mutations co-occurred at very low frequency (1% or less). Whereas, in lesions from progressing patients, mutations in NRAS^Q61^ and BRAF V600 co-occur in up to 30% samples. Another mechanism of resistance to BRAF inhibitor can be an alternate splicing of V600E RNA. This emerges from the data of the biopsies performed on metastatic melanoma patients progressing during anti-BRAF therapy. On 21 patients tested at progression, 10 had either BRAF splice variant or NRAS mutation. In addition, PLX4720 enhances the levels of the anti-apoptotic BCL-2 family member protein MCL-1 in NRAS mutant melanoma cells through enhanced signaling through the MAPK pathway [58].

Primary resistance to BRAF inhibition is present in approximately 5-10% of patients with BRAF mutant melanoma patients including CCND1 amplification in tumors, leading to downstream over expression of Cyclin D1 and enhanced CDK4 expression. CTNNB1 mutations co-occurred pre-therapy and at progression at ~5-10% frequency. Sanger Sequencing (exons 2, 3 and 6) of MEK1 demonstrate, MEK1^P124^ mutations co-occur at low frequency (6/132 pts, ~7%) with BRAF V600 mutations prior to treatment. Rare MEK1 mutations (codons 56, 121, and 203) that are known to reactivate MAPK pathway and be resistant to MEK1 inhibitors have been identified at progression. These might represent pre-treatment markers of resistance which can help to identify patients who are not likely to respond to BRAF inhibitors.

Recent RNA interference screen showed that neurofibromin (NF1) drives resistance to BRAF inhibition [59]. The NF1 gene encodes the tumor suppressor gene that inhibits NRAS activity. Loss of function mutations in NF1 results in the sustained activation of MAPK pathway by increasing RAS signaling, which renders cells resistant to RAF and MEK inhibitors. The study in samples from patients with melanoma who received BRAF inhibitor therapy suggested a role for NF1 in both intrinsic and acquired drug resistance. Whole-exome sequencing of pre-and post-treatment samples identified 4 patients with NF1 mutations in both pre- and post treatment specimens. IHC studies demonstrated that 2 of 5 expressed little or no protein before BRAF treatment. In the remaining 2 of 3 with initial neurofibromin expression, vemurafenib treatment was associated with loss of protein expression [60]. This study provides new insight into melanoma progression and suggests novel therapeutic strategies for mutant BRAF/NF1-deficient melanomas.

Another novel approach to melanoma treatment under investigation is the use of oncolytic viruses (OVs). OVs are native or recombinant viral vectors that mediate host anti-tumor activity through two distinct mechanisms [61]. First, these viruses enter cells and selectively replicate in tumor cells resulting in lytic cell death. Second, the viral infection and dying tumor cells induce a host anti-tumor immune response. To date, the pox and herpes viruses have been most extensively studied in pre-clinical models and in melanoma clinical trials. Vaccinia virus is the prototypical poxvirus and has been extensively used to express tumor-associated antigens and immune potentiating factors as vaccines in a variety of different cancers [62]. Recombinant Vaccinia viruses encoding the human B7.1 T cell co-stimulatory molecule alone or in combination with intra-cellular adhesion molecule 1 (ICAM-1) and leukocyte function associated antigen-3 (LFA-3) was used in a Phase I clinical trial as on OV in patients with unresectable melanoma. In this study the virus was found to be safe with only a few low-grade side effects (fever, fatigue and mylagias) reported. Vaccination resulted in the induction of anti-viral antibody titers and generated gp100-specific T cell responses that correlated with regression of injected lesions. We identified the immunoglobulin-like transcript 2 (ILT2) gene that was significantly down-regulated in T cells exposed to the recombinant Vaccinia vectors using a gene microarray strategy. ILT2 is a negative regulatory protein that inhibits T cells responses and decreases in ILT2 were associated with therapeutic responses in vaccinated patients, suggesting this might be a useful biomarker of clinical response.

An attenuated, recombinant herpes simplex type 1 virus (HSV-1) encoding GM-CSF has been constructed. This vector has demonstrated regression of both injected (oncolytic) and uninjected tumors in murine models. HSV-1 encoding human GM-CSF was constructed and has been named Talimogene Laherparepvec (T-VEC). T-VEC was shown to be safe in a Phase I clinical trial and was tested in 50 patients with locally advanced and metastatic melanoma in a Phase II study [63]. This study further demonstrated limited toxicity, induction of anti-herpes viral titers in all patients and objective regression of tumors in 28% of the treated patients, including regression of distant, un-injected melanoma. Selected patients also had biopsy specimens taken after vaccination which revealed the induction of a MART-1-specific CD8+ T cell response and decreased numbers of CD4 + FoxP3+ regulatory T cells, CD8 + FoxP3+ suppressor T cells and MDSCc in injected lesions. Based on these data, a large T-VEC has been tested in an international, multi-institutional, randomized, controlled phase III clinical trial with results expected in 2013.

In summary, understanding of the molecular drivers of melanoma provides opportunities to target the disease in a rational manner that may ultimately reduce the mortality from the disease. Different signaling pathways have been identified in melanoma with genomic analysis of primary melanoma giving an insight into the molecular drivers of the disease at an early stage in disease progression and include mutations in a variety of genes including well established BRAF, NRAS, KIT, MAPK kinases, and novel genes such RAC1. These genomic changes frequently involve classic oncogenic pathways including the PI3K/AKT/mTOR, RAS/RAF/MEK/ERK and the CyclinD1/CDK4/pRB pathways. Mutations in genes that activate these pathways provide opportunities to therapeutically target the disease. Mutations in BRAF in about 50% of melanomas can be inhibited by drugs that target the RAS/RAF/MEK/ERK pathway including vemurafenib, dabrafenib, and trametinib. Similarly, mutations in NRAS in about 15-18% of melanomas also activate the RAS/RAF/MEK/ERK pathway that can be inhibited by MEK-inhibitors such as MEK 162. Other therapeutic opportunities exist in targeting mutations in ERBB4 present in about 10% of melanoma patients that could be inhibited by lapatinib. Upregulated EphB4 receptor expression present in about 10% of melanomas could be inhibited by nilotinib, and imatinib, sunitinib; and nilotinib inhibit KIT mutations that occur in about 3-5% of melanomas.

BRAF inhibitors have dramatically changed the outcome of patients with advanced disease that contain mutations at the codon V600. However, the responses are not long lasting and result in progressive disease in majority of patients. Significant progress has been made in understanding the mechanisms of the intrinsic and acquired resistance that can be both MEK-dependent and MEK-independent. To overcome MEK-dependent resistance the combination of a BRAF inhibitor (dabrafenib) with a MEK inhibitor (trametinib), has resulted in significant extension of PFS, when compared to dabrafenib as single agent. BRIM 7 trial combining vemurafenib with a potent inhibitor of MEK1/2 (GDC 0973 or cobimetinib) demonstrated a high response rate in melanoma patients’ naïve to BRAF inhibitors. However, adding the MEK inhibitor to vemurafenib-progressive patients, has led to a much less impressive response rate (< 25%). The well-documented immunotherapy approaches for melanoma targeting key T cells immune check points such as CTLA-4 and PD1-PD-L1 have already validated the merits for combining these agents with targeted therapy approaches. Targeted therapy increases the sensitivity of the tumor for immunotherapy by recruiting immune cells into melanoma lesions supporting the merit of combining those two types of therapy to achieve clinical benefits. Other possible approaches to overcome developing resistance are to target other aspects of melanoma biology downstream of BRAF such as cell cycle regulation, tumor metabolism or activation of an immune response. Potential therapeutic strategies include the use of CDK4 inhibitors such as PD-0332991, and inhibitors of glycolytic regulators of tumor metabolism such as inhibitors of pyruvate dehydrogenase kinase or lactate dehydrogenase that inhibit tumor cell glucose metabolism. A cancer drug based on oncolytic virus has also succeeded in a late stage clinical trial giving another agent for multi-modality care.

In conclusion, many mechanisms of resistance have been driving progression during anti-BRAF therapy, likely involve MAP-kinase pathway in most cases, and it is likely that more than one resistant mechanism is present at the same time. It’s difficult, if not impossible, to target all the resistance mechanisms after they have been “activated”, but combination therapy used upfront may prevent resistance (i.e., BRAFi plus MEKi). Combination therapy approaches that incorporate targeted agents (e.g., vemurafenib) and immunotherapy (e.g., ipilimumab, anti-PD1/PDL1) are most promising and are already clinically explored. Despite the remaining challenges, it is certain that rational combination therapy for treatment of patients resistant to BRAF inhibitors represents the future of metastatic melanoma treatment.

## Novel concepts

Melanoma is a complex and heterogeneous disease and tumor may be composed of distinct cell populations with distinct molecular features and varying response to treatment. Inter-and intra-tumor heterogeneity within primary tumours and between primary and metastatic sites as well as developing resistance suggest the need for novel approaches to identify predictive markers closer to patients’ tumor allowing for rational design of combination therapies instead of using a single biopsy assumption. Additional challenge is how to use the knowledge that cancer development and progression depends on immune system and stroma and several areas of new investigations provide promissing insights with regard to development of successful treatments. Other studies including the role of microbiota, mechanisms of rejection and metastasis process are essential for continuous progress in the treatment of melanoma. Thus, it is important that the agents to down-regulate the immune suppression, drugs targeting vasculature or crucial regulators of metastasis complimentary to targeting tumor cells are considered for rational therapeutic strategies.

Humans have co-evolved with microbial partners. We are a composite of species: including bacteria, fungi and viruses. In and on our bodies, microbial cells outnumber the human cells by about 10 fold. In the intestine, the total microbial DNA (the microbiome) may contain 100 times more genes than our ‘own’ human genome. The microbiome is an integral part of our genetic landscape and regulates metabolic functions. The development of the immune system is even dependent on interactions with the commensal microbiota.

Commensal bacteria affect local and systemic inflammation/immunity, influencing skin, mucosal and intestinal immunity, and relative inflammation. In experimental mouse model of subcutaneous transplanted tumor, antibiotics suppress the anti-tumor effect of anti-IL-10R/CpG therapy, decrease inflammatory cytokine production in the treated tumors, and suppress early necrosis of the tumor. Antibiotics treatment also impairs the anti-tumor effect of chemotherapy with platinum compounds such as oxaliplatin [64].

Tissues respond to infection or damage with inflammation and immunity in which all tissue cell types actively participate in order to fight infections, repair damage, and restore tissue integrity and function. Chronic inflammation can promote all phases of carcinogenesis, from initial genetic alterations promoting tumor development, to establishing tumor environment that promotes tumor initiation and progression, and triggering immunosuppressive mechanisms that prevent anti-tumor immune response. This intrinsic inflammation is carried out by epithelial cells, endothelial cells (ECs), fibroblasts, infiltrating hematopoietic cells and other stromal cells that produce cytokines, chemokines and growth factors that affect tumor cells phenotype as well as create micro-environment in surrounding tissues which is responsible for activation of a proinflammatory program. By contrast, extrinsic inflammation is activated by the tissue response to the malignant cells, and it is predominantly mediated by the infiltrating inflammatory cells and regulates tissue rearrangement, angiogenesis and ability of tumor cells to disseminate [65]. Oncogenes such as RAS, BRAF, MYC, RET, Src affect not only tumor cell proliferation and transformation but also production of pro-inflammatory factors such as cytokines and chemokines that activate inflammation by recruiting inflammatory cells and creating environment with reduced anticancer response.

Involvement of different mechanisms and compounds in inflammation is postulated. For example, the signaling adaptor molecule myeloid differentiation factor 88 (My88) downstream of most TLRs and of the IL-1 family is central for the activation of NF-κB mediated innate inflammatory pathways. My88 is required for RAS induction of pro-inflammatory genes through a positive feed-back mediated by IL-1α binding to the MyD88-coupled IL-1R. An IL-1α autocrine loop in oncogenic RAS transformed mouse keratinocytes is required for both the secretion of pro-inflammatory mediators and, in part, for the transformation phenotype [66].

Tumor-associated macrophages (TAM) are a key component of cancer promoting inflammation [67-69] and TAMs are generally skewed in an M2-like phenotype of IL-12 and IL-23 low and IL-10 high with variable capacity to produce inflammatory cytokines [68]. Evidence suggests that the inflammation driven by these cells promotes tumor proliferation and progression, stimulates angiogenesis and lymphangiogenesis, inhibits adaptive antitumor immunity contribute to tissue remodeling and promotes genetic instability. The potential of TAM markers and, more in general, inflammation markers as diagnostic tools to tailor chemotherapeutic and, most important, immunotherapeutic approaches deserves further study [70]. In particular, a distant relative of C reactive protein, PTX3, has emerged as a potential correlate of cancer related inflammation in diverse human tumors [71]. The value of markers of inflammation as predictors of response to immunotherapy in melanoma is also of high importance. Strategies aimed at interfering with TAM recruitment yield encouraging results including re-education with activation of the antitumor activity of these cells [72] or reduction of their numbers. There is evidence that TAM express high levels of PDL1 and therefore can be a target for PD1-PDL1 blocking strategies. Approaches aimed at reducing the numbers of TAM include inhibitors of chemokines and of colony stimulating factor 1 (CSF-1). Recent evidence indicates that trabectedin, an antitumor agent approved in Europe for the treatment of sarcomas and ovarian carcinoma, acts by depleting TAM in the mouse and in man [73]. These data provide proof of principle evidence that targeting TAM offer promise for the development of effective therapeutic strategies.

Ulcerated melanoma is a distinct biologic entity, considering that survival is much lower for same Breslow thin melanomas [74]. Ulcerated melanomas differ from non-ulcerated melanomas in terms of stromal response [75] gene profile signature [76] and a sentinel node immune-suppression status [77]. Analysis of the data from two large phase III trial with IFN as adjuvant therapy for melanoma, the EORTC 18952 (intermediate doses of IFN-α2b) and the 18991 pegylated-IFN (pIFN), demonstrated that ulceration is predictive for the efficacy of adjuvant IFN/pIFN therapy. In both of these trials patients with ulcerated melanoma have an advantage in relapse free survival (RFS), in distant metastasis-free survival (DMFS) and in OS according to the stage (IIb & III – N1) versus the non ulcerated melanoma patients [78-80]. This advantage disappears in N2 patients. The EORTC Study 18991, after a 7.6 years follow up, confirmed the data of advantage in this subgroup of patients, by a 42% Relative Risk reduction in overall survival [81]. Also in the SUNBELT trial only patients with ulcerated melanoma benefitted from IFN treatment [82]. Even from the Wheatley meta-analysis of IFN as adjuvant therapy in melanoma, ulceration emerged as the advantage for melanoma patients, so that ulcerated melanoma can be considered a distinct biologic entity because only ulcerated melanoma is sensitive to IFN, while non ulcerated melanomas seem to not benefit at all from adjuvant IFN therapy [11]. The EORTC 18081 trial in ulcerated stage II melanoma patients evaluates prospectively the benefit of adjuvant pIFN in this setting.

Intratumour heterogeneity (ITH) is variability within a tumour. Clinicians, particularly pathologists, have understood morphological differences in cancer for decades. ITH includes variability within primary tumours and between primary and metastatic sites

In the same patient. ITH can be viewed in an evolutionary framework and has been identified clearly in renal cell carcinoma (RCC). A fundamental question for personalised medicine is whether the putative driver identified from tissue “x” at time “y” really does drive the metastatic disease in the patient in the clinic. Furthermore, do image-guided biopsies of large renal tumours represent the entire primary, never mind the burden of metastatic disease? Single biopsy assumptions are that the tumour somatic/transcriptomic landscape is represented and provides robust prognostic and predictive biomarkers of outcome and that mutations are ubiquitously present in every region of a tumour and can be used for sequencing analysis to stratify patients for therapeutic trials. But these assumptions are not always valid. The E-PREDICT study [83] compared mutations between primary RCC tumours and metastases, showing that 65% mutations are heterogeneous and are not present in every biopsy in the patients reported.

There may be evidence for ITH in melanoma; polyclonality of BRAF mutations in primary and metastatic samples has been shown and in samples from patients resistant to Vemurafenib, persistence of BRAF mutations has been shown. These melanomas, however, have acquired NRAS mutations or PDGFRβ overexpression. Furthermore, distinct subclones identified with IHC within a metastasis progressing on vemurafenib have been studied: one remained BRAF mutant; the other acquired NRAS mutation in addition. Re-sensitisation of patients to BRAFi therapy after time off treatment is another interesting observation that may be explained by ITH [84].

Concluding, intratumour heterogeneity has been around for decades, and in RCC, as in other cancers, it can be demonstrated using multiple methods. Evidence for ITH in melanoma is perhaps less convincing at present, but it is plausible though that it may be relevant to drug resistance. Further study is needed (e.g. detailed longitudinal tissue collection), to investigate if ITH may impact on therapeutic strategies.

Stereotactic radiotherapy (SRT) can be considered the best local treatment for brain metastases, controlling nearly all treated lesions for at least several months. Since the advent of SRT over a decade ago, the pattern of survival has changed to longer and more likely to succumb to extracranial disease than to brain metastases. Melanoma brain metastases have always been considered a “death sentence” with less than 6 months’ median survival for most subgroups of patients. Fortunately, questions about the risks and biology of brain metastases (e.g., molecular pathogenesis, tumor-host relationships, site of primary and its environmental and genetic features) have started to see answers, and much new information is expected from collaborative networks that have been developed to answer these and other questions about biology and management of brain metastases. In 296 patients with resected brain met(s) BRAF and NRAS status was determined, and they were evaluated between Jan 2005-Dec 2011 (before BRAFi, MEKi therapies) [85]. In terms of difference in median age, BRAF wild-type (WT) patients were 66 years, while BRAF (all comers) and NRAS mutated patients were mid-50s. Of 99 pts (33%) with symptomatic brain metastases, 13 (17%) were WT Type, 21 (40%) were NRAS mutated and 65 (39%) were BRAF mutated. The outcome of the patients with melanoma brain metastases resulted different according to the mutational status. Wild-type patients had a better prognosis compared to mutated patients (any mutation/WT HR 1.88, p = 0.04), and the pair wise comparison evidenced a better prognosis for BRAF-mutated patients than NRAS-mutated pts (BRAF/WT HR: 1.75; NRAS/WT HR: 2.31). The subgroup analysis showed that the differences in outcome among the three groups of patients could be lessened by locally ablative treatments, although there was a persistent advantage for WT pts. Recent single-patient case reports have been informative and led to formal testing in Phase II trials. Thus, a report by Rochet et al. showed a patient with a BRAF-mutated melanoma who failed to benefit from ipilimumab but experienced a response of brain metastasis to vemurafenib [86]. At the 2010 ASCO Congress the preliminary data on dabrafenib, another BRAF inhibitor similar to vemurafenib, in the treatment of melanoma patients with brain metastases were presented [4]. Of 10 treated patients, there were 3 CR, 5 PR, 1 MR and 1 SD, for an 80% BORR (best objective response rate). These data provided the basis for the BREAK [“BRAF E and K” mutations]-MB [Melanoma Brain] trial, enrolling melanoma patients with brain metastasis, harboring V600 mutation, previously treated or untreated with radiotherapy, to be treated with dabrafenib. For patients untreated with radiotherapy, brain OR was 39% (E600E), and 7% (V600K), while for previously treated brain OR was 31% (V600E) and 22% (V600K).

Ipilimumab was tested in two clinical trials to identify safety and overall survival of patients with advanced melanoma and brain metastases. Dose/schedule were identical at 10 mg/kg q 3 wks × 4, followed by 10 mg/kg q 12 weeks until relapse or intolerance. The two trials are the CA184-042 trial and the CA184-045, an open-label, expanded-access treatment protocol. Patients who qualified for these trials experienced an approximately 20%-25% durable benefit at 2+ years of follow-up, with rare progressions beyond this time.

The NIBIT trial, associating ipilimumab at the dosage of 10 mg/kg with fotemustine, treated melanoma patients with brain metastases. Results were 40% overall disease control rate, and 50% CNS disease control rate, with a good toxicity profile. The role of fotemustine is unknown and is being studied in an ongoing randomized trial but the drug is unavailable in the U.S. Although ipilimumab has been combined with cytotoxic agents such as dacarbazine and temozolomide (TMZ), the benefits of each component and their interactions could not be assessed from the trial designs. Toxicities may limit the further exploration of such combinations, and there is currently greater enthusiasm for combinations of checkpoint blockade with molecularly targeted agents. Nevertheless, some of the current immunotherapeutic strategies are investigating the use of selected chemotherapies to transiently lymphodeplete the patient in an effort to alter T cell subsets and stimulate them with increased levels of homeostatic cytokines.

Brain vascular endothelium promotes metastatic growth, invasion, altered architecture and function. Fewer vessels are more permeable and viable or hypoxic tumors produce VEGF by hypoxia-related gene expression, and tumor-associated stromal cells, e.g., fibroblasts, contribute to support metastases. Microglia and astrocytes may also be protective. Blood–brain barrier (BBB) histologically is composed of ECs, pericytes, astrocytic perivascular endfeet and makes an active process of blocking, because molecules can’t pass through tight junctions, but must pass through cells. An active transport system regulates the passage of small hydrophilic molecules, while large hydrophilic molecules are excluded or admitted via receptor-mediated endocytosis [87]. The barrier breaks down in tumors over 1–2 mm, and heterogeneity of BBB within/around tumors may protect tumor against effective radiotherapy. Various therapeutic strategies have been directed to overcome tumor-protective BBB as Lipoprotein-related protein-1 targeting of cytotoxic agents, inhibitors of drug-efflux pumps/multi-drug resistance and use of drugs that cross intact BBB, e.g., fotemustine, TMZ, and anti-angiogenic therapies that include Abs and small molecules.

Molecular mechanisms are common to various malignancies. Between these the most important are VEGF, hypoxia-related genes, WNT signaling, EGFR-related, Immune/inflammatory (TGF-β expression, CD4, NK cells, B7-H3).

Molecular mechanisms of particular interest for melanoma are STAT3 that regulates angiogenesis gene expression VEGF, and results in improved brain metastases with respect to primary tumor, so the level of gene expression are associated with potential for brain metastasis. Rx synergy of STAT3 block, and AKT that is hyperactivated in human brain metastases without PTEN loss promoted brain metastasis was modeled in genetically-engineered mouse. AKT may be activated by secreted product of astrocytes, and can have therapeutic implications [88].

Nab-paclitaxel is the first nanotechnology-derived agent approved for the treatment of breast cancer. This formulation exhibits linear pharmacokinetics over a clinically relevant dose range 3, which means predictable drug exposure with dose modification. Compared with solvent-based paclitaxel, nab-paclitaxel exhibits linear pharmacokinetics, ~10-fold increase in Cmax and ~3-fold higher AUC (area under the curve) of unbound paclitaxel, potential binding to albumin-binding proteins and shows enhanced transport across endothelial cell monolayers, with a 33% higher paclitaxel concentration in tumor xenografts [89].

Melanoma cells express high levels of SPARC (serum protein acidic and rich in cysteine), an albumin-binding protein that may facilitate delivery of nab-paclitaxel to tumor cells. SPARC, also known as Osteonectin and BM-40, is a 43-kDa glycoprotein that interacts with extracellular matrix proteins. Normal functions of SPARC may include tissue remodeling, angiogenesis, cell motility, mineralization of bone and cartilage, and embryonic development. SPARC is overexpressed in many cancers, especially in cells associated with the tumor stroma and vasculature, and it may play a role in tumor progression in different cancer types.

There is a direct correlation of SPARC expression and OS in patients with pancreatic cancer who received nab-paclitaxel plus gemcitabine, as evidenced in a Phase I/II Trial, with a median OS of 17.8 months in the high SPARC expression group *vs.* 8.1 months of low SPARC expression group. Although high SPARC expression is typically a poor prognostic factor, it actually predicted an improved response to nab-paclitaxel in terms of OS.

In phase I and II studies nab-paclitaxel produced promising efficacy, differently from cremophor-paclitaxel or docosahexaenoic acid-paclitaxel, that produced limited clinical benefit. The phase III trial randomized 514 pts with chemo-naïve metastatic cutaneous melanoma to be treated with nab-paclitaxel or dacarbazine. Primary efficacy endpoint was PFS while secondary efficacy endpoints were OS, ORR and DCR. PFS of nab-paclitaxel group resulted 4.8 months, while PFS of dacarbazine group resulted 2.5 months. The advantage was also obtained in OS with 12.8 months for nab-paclitaxel group *vs.* 10.7 months for dacarbazine group. The BRAF-mutational status did not change the outcome of nab-paclitaxel group. Improvement in PFS with nab-paclitaxel occurred in all patients regardless of age (<65, ≥65 years), region, BRAF mutation status, and baseline LDH (lactate dehydrogenase) level. In particular, for patients with the most advanced melanoma (M1c), nab-paclitaxel produced significantly longer PFS (HR: 0.734; 95% CI: 0.558–0.965, P = 0.028) compared with dacarbazine. The treatment showed a good safety profile [90].

Nab-paclitaxel is the first taxane and first single-agent chemotherapy in 35 years that demonstrated superiority compared with dacarbazine with a near doubling of median PFS, a 44% improvement in disease control rate (DCR), and a trend towards OS benefit in chemo-naïve patients with metastatic melanoma. Future perspectives could consist in the combination of nab-paclitaxel with newly approved biologic/immunologic therapies (BRAF inhibitors in BRAF mutated patients or ipilimumab independently of BRAF mutational status).

Understanding how tumor rejection occurs under a given immunotherapy is one of the primary goals of clinical research in order to optimize existing immunotherapy and development of new therapies. Through multiple cancer immunotherapy molecular monitoring, a repetitive phenomenon has been observed that the activation of different factors as STAT1/IRF1, allograft inflammatory factor 1, IL-15, IL-6, Granzyme A, B, K, Perforin, CCL4 (MIP-1b), CXCL10/IP-10, CXCL9/Mig, and/or CCL5 are necessary for tumor rejection. Nevertheless, tumor rejection is a multifactorial process involving host’s genetic background, environmental/hidden factors and tumor genetics.

In order to dissect contribution of each components to the clinical outcome of melanoma patients undergoing adoptive T cell therapy TILs from 142 patients enrolled in adoptive T cell therapy trials, 112 parental melanoma metastases and 15 melanoma cell lines derived from melanoma metastases were analyzed. Global transcriptome analysis of the tumors sample using gene expression array analysis, micro RNA profiling as well as genome wide single nucleotide polymorphism (SNP) association analysis of advanced melanoma patients who achieved CR and of those that progressed (NR) was performed. The results demonstrate that TILs that lead to complete tumor regression in patients share a gene signature distinctive of memory cells. Pathway enrichment analysis revealed down regulation of genes associated with differentiation as well as enhanced proliferation function in CR compared to NR patients. The data underscore the notion that these T cells are less differentiated but have enhanced proliferative phenotype consistent with recently published data [91]. These memory T cells which were designated memory stem T cells (T_SCM_ cells) share the similarity of naïve phenotype comparing with effector T cells that have more proliferative properties and prolonged survival post adoptive transfer in preclinical model.

Significant segregation of responders from non-responders was also observed based on the gene expression profile of the tumor cell lines, leading to the conclusion that immune responsiveness is at least in part dependent upon the biology of cancer cells [92].

Comparison of the miR profile in complete responders versus nonresponders identified 13 distinct miRs between CR and NR, suggesting that clinical outcome can be regulated at the epigenetic level. Genome-wide scan analysis of germline SNPs based on allele frequency identified 5 loci in IRF5 gene with global significance that were associated with clinical outcome suggesting that genetic polymorphism is an important component determining tumor rejection.

Concluding, immune responsiveness is at least in part is dependent upon the genetic background of the host and the biology of cancer cells. The finding that adoptive T cells have enhanced proliferative capacity and can sustain the function of memory and effector T cell subsets has considerable implications for the design of T cell–based therapy to target intracellular pathogens and cancer.

Sentinel node biopsy has completely changed the surgical approach to melanoma patients, it has permitted to stage properly the patients and it has opened new ways of researches on the biology of tumor metastases. By the newer technologies a computer-assisted probe with adjustable collimation has been developed. By this machine, energy windows can be calibrated by computer, according to the energy spectrum emerging from each patient. In this way, the possibility to optimize photoelectric/compton efficiency reduces the dimensions of the smallest detectable node, possibly reducing the false negative sentinel lymph node biopsies and the possibility of errors of surgeons and nuclear radiologists. Conventional SPECT imaging is a Single Photon Emission Computed Tomography [93]. The Free Hand SPECT is a real extension of gamma probe for 3D imaging that could be easily positioned in the operative room. By the elaboration of the computer, Free Hand SPECT gives real-time view with 3D reconstruction, combined with traditional acustic signal. By preliminary tests, this device seems useful in cases of SN near the melanoma, deep nodes, over-weight patients and to confirm that no hot nodes remains in site at the end of the procedure.

Sentinel node (SN) is the first point of contact between tumor-associated antigens and the adaptive immune system, and is the site where dendritic cells (DCs), the professional antigen presenting cells (APCs), are expected to present tumor antigens to naive T-cells generating specific responses against tumors. SNs show down-regulation of APCs, because DCs show little or no formation of dendritic processes. Plasmacytoid dendritic cells (p-DCs) have been described in primary melanoma lesions and p-DCs represent the main source of IFN-α. There is a possible role of p-DCs in priming naive melanoma-specific CD8+ T-cells as well as in IFN production. The infiltration of p-DCs has a prognostic value in primary breast cancer, as its presence correlates with adverse outcome. p-DCs are rarely detected in lymph nodes of healthy subjects. p-DCs have been demonstrated in SN of melanoma patients and they accumulate around melanoma nests in situ, and their percentage increases in presence of melanoma metastasis [94]. Langherans Cells (LCs) represent the major DCs subset in melanoma SN; they have low expression of CD83, CD80, CD86, HLA-DR markers, and CD83 positive LCs are lower in SN positive than in SN negative melanoma patients [95]. These data could support the hypothesis of new therapeutically effective strategies, in order to reverse the immune suppression of tumor related nodes, possibly inducing an effective immune response against the tumor.

Electrochemotherapy (ECT) is a new therapy for cutaneous metastases that uses reduced doses of antineoplastic drug in association with electroporation to create a difference of potential in the cell membranes that opens the pores by which the chemotherapy agent enters inside the cell and gives a cytotoxicity-induced DNA break. Electrochemotherapy is effective, with 85% Objective Response (OR), 73.7% CR and 11.1% Partial Response (PR). The procedure is safe; the side effects are absolutely slights with only erythema, edema, necrosis, discromia and with no grade III-IV toxicity. Possible mechanisms of action are cytotoxicity on cancer cells that give necrosis and apoptosis of cancer cells by electric pulses. Immunological mechanisms could play a role, as 80% of tumors cures were achieved in immunocompetent mice and none in immunodeficient mice. Moreover, it has been demonstrated a reduction of epidermal LCs at biopsies performed at day 7 post-ECT, an increase of epidermal LCs at day 14 post-ECT, an increased amount of peri-tumoral dermal DCs CD1c+, HLA-Dr + post-ECT and the appearance of peri-tumoral CD83+ DCs in the dermis post-ECT [96]. All together these data indicate that ECT could induce migration and activation of LCs. It could be hypothesized that ECT triggers initial and local immune responses in treated lesions and it could also be hypothesized to modulate this scenario and enhance the potential ECT actions [97]. According to these immunological stimulations, a trial of ECT plus ipilimumab has been planned.

Liver metastases are a typical manifestation of progression of disease of uveal melanoma, but even cutaneous melanoma demonstrates predominant hepatic metastases in some cases. Liver anatomy is favorable to vascular isolation, as tumors receive the majority of their blood flow from the hepatic artery, and hepatic metastases are often the cause of death of the patient. Isolation and arterial perfusion of the liver eliminates or significantly reduces systemic toxicity, and allows higher doses of toxic chemotherapy. Isolated liver perfusion began in the 1960’s, using an open, operative technique. The advent of advanced vascular catheters and an extracorporeal venous hemofiltration system have allowed development of a minimally invasive, percutaneous technique. A phase I study of Hepatic Arterial melphalan Infusion and Hepatic Venous Hemofiltration using percutaneous catheters in patients with unresectable hepatic malignancies enrolled 12 patients treated at dose of 2.0 mg/kg then dose escalation (2.5, 3.0, 3.5 mg/kg) [98]. Planned treatment course included 4 treatments separated by 4 weeks, and 3 mg/kg was the maximum tolerated dose. Dose limiting toxicities were neutropenia and thrombocytopenia. Overall radiographic response rate was 30%. Among the 10 patients with ocular melanoma, a 50% overall response rate was observed, including two complete responses. With chemosaturation therapy using percutaneous hepatic perfusion (CS-PHP) it is possible to use dosages of melphalan 12 times higher than the dose used for the systemic treatment of multiple myeloma. At the 2010 ASCO Congress phase III data were presented, in which 93 pts with Stage IV melanoma - Liver-only/liver predominant metastases (stratified as cutaneous or uveal) were randomized to receive CS-PHP or best alternative care. Significant improvement in the primary endpoint was obtained with an extension of median PFS by 6.6 months (HR 0.30 (p < 0.0001). There was consistent improvement in secondary endpoints, but the crossover design of the trial precluded evaluation of an impact on OS [99]. Expected bone marrow toxicities of melphalan were the main side effect. New developments for CS-PHP include second generation filters, with improved drug extraction efficiency. First generation filters have a clinical extraction efficiency of 77%, whereas in bench-top experiments, second generation filters have extraction of approximately 97%. Demonstration of improved extraction and possibly decreased systemic drug exposure and toxicity are pending in ongoing studies.

## Bridge between melanoma and colo-rectal cancer: biology of tumor microenvironment

Discovery of specific mutations leads to development of clinically useful inhibitors of critical signaling pathways but usually is limited to specific type of tumor. However, the importance of the immune response and the role of microenvironment in cancer development, progression and metastasis has been recognized as potential therapy target to improve cancer patients survival in many tumor types, including melanoma. For example, the critical role of the microenvironment in the growth of cancer is supported by the role and the therapeutic potential of angiogenesis. Current data indicate that secreted stromal factors that can mediate drug resistance and predict response to treatment based on microenvironmental factors could be shared between various tumors. Thus, promising studies of microenvironment and immune response in different type of tumors can benefit melanoma and *vice versa* as discussed in this section.

A lack of balance of host tumor interaction is due to numerous interactions and is mediated by at least three distinct escape mechanisms involving:1) alteration of genomic and phenotypic features of tumor cells themselves (expression of tumor antigens, somatic mutations, up-regulation of anti-apopototic molecules, 2) the tumor micromilieu (melanoma cells interact with the microenvironment in bidirectional manner through molecular signals that modulate malignant phenotype), as well as 3) immune cells that can either become immunosuppressed or reject the tumor cells. Cancer cells interact with stromal cells by production of certain factors affecting fibroblasts or ECs resulting in a cascade of events leading to progression For example, melanoma cells secrete growth factors such as FGF-β PDGF, and TGF-β that affect angiogenesis and stroma formation by inducing proliferation of fibroblasts and ECs. The melanoma cells can also produce VEGF resulting in ECs growth. In return, EC produce chemokines resulting in melanoma cell migration (e.g., CCR4) via receptors found on melanoma cells that can contribute to drug resistance to cancer therapy. HGF secretion by stromal cells was shown to be responsible for resistance to targeted therapy including BRAFi. The impact of interactions of tumor cells and stroma is increasingly recognized as important target to individualize therapy for melanoma patients.

An altered interplay between immune system and tumor cells towards inflammation that promotes carcinogenesis represents a major cause of tumor progression. This process includes downregulation of MHC molecules, secretion of immune suppressive cytokines (VEGF, IL-10 and TGF-β, recruitment of inhibitory immune cells (e.g., Treg and MDSCs) and others. Loss or downregulation of HLA class I antigens often occurs in tumor cells and is associated with disease progression, reduced survival of patients as well as lack of response to T cell-based immunotherapies. Although, metastasis expressing HLA class I antigens in melanoma patients undergoing immunotherapy can regress immunotherapies can partially generate HLA class I loss variants causing progression. The HLA class I abnormalities are mediated by an impaired expression and functional defect of distinct components of the antigen processing machinery (APM) resulting in a decreased HLA class I surface expression, an altered antigen repertoire as well as a reduced recognition by CD8^+^ cytotoxic T lymphocytes (CTL) [100]. The molecular mechanisms of defective APM components expression may include structural alterations in β_2_-microglobulin, the MHC class I heavy chain (HC) as well as the peptide transporter TAP, but these are rare events in melanoma. In most cases, deregulation of the APM components that are found, occurs at the transcriptional or post-transcriptional level or they it is regulated by epigenetic mechanism. These data suggest that the deregulation of HLA class I APM components is a major cause of deficient HLA class I surface expression in melanoma. The challenge in the future is the identification of regulators of APM components, which are able to specifically modulate their expression. Furthermore, in addition to aberrant expression of classical HLA class I antigens, the non-classical HLA-G is often discordantly expressed in renal cell carcinoma and melanoma [101]. HLA-G expression caused a reduced sensitivity of NK- and T cell-mediated cytotoxicity. We have recently identified HLA-G-specific microRNAs, which might serve as a tool for prognosis and therapy selection due to an inverse expression between HLA-G and microRNA expression, which appear also to correlate with disease progression.

Targeting antigens expressed by tumors in their microenvironment was evaluated in a multi-step process that focused on patients with non small cell lung cancer (NSCLC) or prostate cancer who received autologous tumor vaccines. Protein arrays (ProtoArray) were used to detect the difference in pre- and post-treatment antibody responses. Since autologous tumor was not available for most patients, gene expression profiles of established cell lines were used. Studies with NSCLC patients exhibiting a CR (n = 3) or stable disease (n = 1) found a common pattern of antibody responses against antigens that were shared by the 13 NSCLC cell lines [102]. Next IHC analysis of normal and lung cancer tissue specimens from tissue arrays were evaluated for expression of the proteins identified as targets of a post vaccine antibody response. For a panel of three antigens studied, malignant tissue, but not normal tissue, stained strongly positive for expression of markers tested. These data argue that the ProtoArray technology can be used to assess antibody responses against targets relevant to cancer immunotherapy.

Next a novel immunotherapy strategy employing autophagosome vaccines that contain defective ribosomal products (DRiPs) and short-lived proteins (SLiPs) was reviewed. DRiPs and SLiPs are the center of MHC class I antigen processing pathway, and link the immunosurveillance of viruses and tumors. DRiPs enable the immune system to rapidly detect alterations in cellular gene expression with great sensitivity. DRiPS and SLiPS are rapidly degraded by proteasome, transported to the endoplasmic reticulum, bound by MHC Class I and transferred to the cell surface where they are postulated to contribute the majority of peptides decorating the surface of tumor cells. Proteosome blockade shunts DRiPS and SLiPS to the autophagy pathway and by blocking a degradative step, autophagosomes can be harvested and used as a vaccine. In preclinical studies, an autophagosome vaccine was more therapeutic than a “Gold Standard” GVAX Vaccine in a 3 day-established 3LL tumor model. Additional studies documented that autophagosomes from one unique sarcoma can prime an immune responses against other independently-derived syngeneic sarcomas and can provide a significant level of protective immunity (challenged 14 days post vaccination) in 8 of 9 tumor combinations tested. In contrast, whole tumor cell vaccines only provided protection from a tumor challenge when the challenge was identical to the tumor used as a vaccine [103]. These findings broke a paradigm with chemically-induced sarcomas that had stood for more than 50 years. As such we consider it to be a promising strategy to move into patients. Thus, an Autophagosome Cancer Vaccine derived from two human cancer cell lines, one of mixed squamous/adenocarcinoma and another adenocarcinoma, was developed. This vaccine contains at least six of the tumor antigens prioritized by NCI, agonists for TLR 2, 3, 4, 7 and 9 as well as HSPs. An NCI-funded randomized multicenter phase II trial of cyclophosphamide with allogeneic non-small cell lung cancer (NSCLC) DRibble vaccine alone or with Granulocyte-Macrophage Colony-Stimulating Factor (GMCSF) or Imiquimod for Adjuvant Treatment of Definitively-Treated Stage IIIA or IIIB NSCLC will start in early 2013. A similar phase II DRibble vaccine trial in breast cancer will start later in 2013.

For optimal therapeutic vaccination against cancer concentrated antigen delivery (DNA, RNA, SLP) with appropriate adjuvant is crucial. Synthetic vaccines allow rational vaccine design. Favoured cancer target antigens are involved in cancer initiation, progression and/or metastasis. Example: oncogenic proteins E6 and E7 of high risk HPV. Long peptide vaccines harboring both CD4 and CD8 T cell epitopes and requiring DC processing are efficient and were found capable of causing >50CR or PR in patients with high grade vulvar intraepithelial neoplasia (VIN), caused by HPV16 [104,105]. DNA prime/long peptide boost may be considered. Processing route of SLP appears to differ from that of proteins. Further improvements are seen by adding pegylated type I Interferon or TLR ligands but especially by conjugating TLR ligands to the long peptides. For maximally effective cancer treatment combination treatment such as long peptide vaccination with chemotherapy or irradiation and inhibitors of checkpoint control monoclonal antibodies (CTLA-4 blocker, PD-1, PD-L1 blockers, anti-IL6 (R), anti-IL10 (R), anti-TGF-β (R) and other immunomodulators) could be applied. Reduce toxicity of the monoclonal antibody treatments by local delivery in slow release formulation close to tumor-draining lymph nodes is observed. Adoptive transfer of cancer-specific T cells is best combined with optimal vaccination.

Malignant Mesothelioma (MMS) is a rapidly progressive lethal tumor and the incidence is steadily increasing worldwide. An actual “epidemic” is expected over the next 10–15 years. No standard treatment significantly improves prognosis of MMS patients. Median OS is 12 months (ranged from 6 to 18 months) from diagnosis. Survival in pretreated patients is even poorer. The second-line therapy is undefined and enrollment in clinical trial for fit patients is encouraged. Immune therapy can be considered a good option of treatment, considering evidence demonstrating that lymphocytic infiltration of pleural mesothelioma correlated with a better survival. Consistently, it has been observed that patients with high level of tumor infiltrating T cells have an overall survival much longer than the patients with a low rate. Along this line different immunotherapeutic agents have been tested in MMS patients but with disappointing results. Anti-CTLA-4 mAb potentiate the anti-tumor immune response. The anti-CTLA4 mAb ipilimumab significantly improves the survival of metastatic melanoma patients (20% long-survivors to 4-yrs) and it is currently in clinical development for other indications. The anti-CTLA-4 mAb tremelimumab has been extensively tested in different tumor types, and it is currently being investigated for different indications. Both ipilimumab and tremelimumab can induce durable stabilization and clinical response (even after initial disease progression).

The MESOT-TREM-2008 Study is a second line, single arm, phase II study with the anti-CTLA-4 mAb tremelimumab in chemotherapy-resistant advanced malignant mesothelioma [106]. The aim of the study was to investigate safety, tolerability, clinical and immunologic activity of the drug in this cancer. Primary end-point was objective response rate, and secondary end-points were safety, disease control rate (CR + PR + SD), PFS, OS, and immunologic activity. From May 2009 to January 2012, 29 advanced MMS patients were enrolled. Results suggest that tremelimumab can be active in MMS patients, and can induce durable stabilization of disease, warranting further investigation. The AEs observed in this study are consistent with tremelimumab safety profile in other indications and treatment associates with major changes in T cell subpopulations. In light of these encouraging results, the Study MESOT-TREM2012 is currently ongoing to explore a different schedule of treatment with tremelimumab in refractory MMS patients.

Melanoma comprises multiple clinical forms that arise and develop through different pathogenic pathways, and understanding pathophysiological implications of specific genes associated with metastatic lesions is crucial to identify new prognostic and therapeutic targets. Consistent with the contribution of VEGF and integrin VLA-4 to prometastatic effects of IL-18 in experimental melanoma models [107], we studied genes associated to melanoma cells’ ability to form metastasis under IL-18 effects, and analyzed their expression in primary and metastatic lesions from melanoma patients. We verified that human melanoma cell lines with IL-18R/VEGF/VLA-4 phenotype produced a higher metastasis number in nude mice than those melanoma cell lines not using this prometastatic pathway. Moreover, distinct signature genes for melanoma cell lines with and without IL-18R/VEGF/VLA-4 phenotype were also identified under basal and IL-18 treatment conditions, through a genome-wide transcriptional comparison. Next, we performed a hierarchical cluster analysis of transcript patterns from a collection of metastatic lesions from advanced stage melanoma patients. Interestingly, all the studied melanoma metastases expressed signature genes from untreated melanoma cell lines irrespective of their IL-18R/VEGF/VLA-4 phenotype. However, only cutaneous metastases and a third of lymph node metastases expressed signature genes from the specific transcriptional response to IL-18 of melanoma cell lines with, but not without IL-18R/VEGF/VLA-4 phenotype. Furthermore, most of studied cutaneous primary melanoma lesions also expressed signature genes from IL-18-treated melanoma cell lines with IL-18R/VEGF/VLA-4 phenotype, irrespective of their Breslow indices, suggesting that IL-18R/VEGF/VLA-4 phenotype is already operating at early stage melanomas.

Results of this study demonstrated the occurrence of metastatic lesions with and without IL-18-dependent genes, suggesting that human melanoma metastasis development occurred via inflammation dependent and independent mechanisms. Signature genes from melanoma cell lines with and without IL-18R/VEGF/VLA-4 phenotype may serve as functional biomarkers of melanoma predisposition to prometastatic effects of IL-18. In this regard, melanoma lesions overexpressing those specific genes from untreated (TGFBI, FEZ1, SLC20A1, POLM, GPKOW) and IL-18-treated (UBE2C, UMPS, IQCE, PFAS, PPAT, PTPLAD, ZBTB9) highly IL-18R/VEGF/VLA-4-expressing melanomas, may be susceptible to prometastatic effects of IL-18, and therefore, should not be treated with this cytokine and other immunotherapies increasing inflammation. Conversely, those genes from highly (CD52, PRSS23, TMEM42) and low (CAPN3, A2M, ARID4A, CNOT6L, CYB561) IL-18R/VEGF/VLA-4 expressing melanomas not expressing IL-18-dependent genes, may be helpful to detect IL-18-unresponsive melanoma lesions, and therefore, better candidates for immunotherapy [108,109].

Reported signature genes allowed for first time the classification of metastatic melanoma lesions according to IL-18R/VEGF/VLA-4 phenotype and IL-18-dependent gene expression. These genes may have diagnostic value as pathogenic biomarkers and therapeutic targets of melanoma predisposition to prometastatic effects of IL-18. At present, it is unknown if IL-18R/VEGF/VLA4 phenotype is necessary and sufficient for the determinism of metastasis in melanoma patients. However, many of the genes associated to IL-18R/VEGF/VLA4 phenotype are candidates for involvement in pathogenic mechanisms of melanoma metastasis based on their known function in melanoma and other malignancies. Therefore, these genes may also be a source of potential targets for the specific treatment of metastases developed via IL-18-dependent and independent mechanisms.

Traditionally, the anatomic extent of the tumor burden in colorectal cancer (CRC) and other type of solid tumors has been the most important prognostic factor. Data on the tumor burden (T-stage), combined with the presence of cancer cells in draining and regional lymph nodes (N-stage) and evidence of metastases (M-stage), amalgamate to provide tumor staging (AJCC/UICC-TNM classification). TNM stages estimate patient post-operative outcome and the rationale for adjuvant therapy. Despite the prognostic power of this staging system, it is becoming recognized within the cancer community that clinical outcome can significantly vary among patients within the same stage. The current classification provides limited prognostic information, and does not predict response to therapy. Advances in this field have alluded to the importance of the immune prevalence within the tumor microenvironment. Histological analysis of human tumors, in particular CRC, has highlighted the importance of the combination of immune variables. We have previously described these major immune parameters, associated with survival in colon cancer, as the “immune contexture” [110]. The immune contexture is defined as the type, functional orientation, density and location of adaptive immune cells within distinct tumor regions [111,112]. A strong lymphocyte infiltration has been reported to be associated with an anti-tumor response and improved clinical outcome. This correlation between the prevalence of tumor infiltrating immune cells and patient outcome has been well documented in melanoma, ovarian, head and neck, bladder, breast, urothelial, colorectal, renal, prostatic, and lung cancer. The majority of studies have demonstrated that high densities of CD3+ T cells, CD8+ cytotoxic T cells and CD45RO + memory T cells are associated with a longer disease free survival (DFS) and/or improved overall survival (OS) [111].

Derived from the immune contexture, a simple and powerful immune-classification has been termed the “Immunoscore”. The Immunoscore (I) is based on the numeration of two lymphocyte populations (CD3/CD45RO, or CD3/CD8 or CD8/CD45RO) quantified within the center of the tumor (CT) and invasive margin (IM). These parameters provide a scoring system ranging from Immunoscore 0 (I0), which has low densities of both cell types in both regions; to Immunoscore 4 (I4), having high densities of both cell populations in both regions. The prognostic value of using these immune criteria was demonstrated in patients with early stage CRC (AJCC/UICC TNM stage I-II CRC) to predict survival and relapse. The five Immunoscore groups were associated with dramatic differences in DFS and OS (P < 0.0001) [113]. In large cohorts of patients at all cancer stages, Cox multivariate analysis shows that tumor progression and invasion is statistically dependent on the Immunoscore. In patients who did not relapse, the density of CD8 infiltrates was inversely correlated with T stage, whereas in patients with recurrence the number of CD8 cells was low, regardless of the T stage of the tumor [114]. Thus, evidence supports the notion to introduce immunological biomarkers, implemented as a tool for the prediction of prognosis and response to therapy. Incorporating the Immunoscore into traditional classification could result in a greatly improved prognostic and potentially predictive tool [114-116]. To be used globally in a routine manner, evaluation of a novel marker should have the following characteristics: validated for routine testing in clinical laboratory, meeting regulatory standards of analytical validity, simple, inexpensive, fast, robust, accurate and reproducible, quantitative, and preferably pathology-based. The Immunoscore fulfills characteristics of clinically useful assay.

In an effort to promote the Immunoscore in routine clinical settings, an international task force was initiated. The working group, composed of international expert pathologists, oncologists and immunologists, identified a strategy for the organization of worldwide participation by various groups for the validation of the Immunoscore. The purposes of the Immunoscore worldwide task force are, to demonstrate the feasibility and reproducibility of the Immunoscore, to validate the major prognostic power of the Immunoscore in routine for patients with colon cancer stage I/II/III, and to demonstrate the utility of the Immunoscore to predict stage II colon cancer patients with high risk of recurrence. Twenty-three (23) international expert centers agreed to participate in this visionary enterprise. These participants represent 23 Centers Worldwide from 17 countries including Asia, Europe, North America, Australia, and Middle East. The outcome of this international validation effort may result in the implementation of the Immunoscore as a new component for the classification of cancer, designated as TNM-I (TNM-Immune).

## Competing interests

PAA has consultant and advisory role for Bristol Myers Squibb, Merck Sharp & Dohme, Roche-Genenetech, Glaxo Smith-Kline, Amgen, Celgene, Novartis. He received honoraria from Bristol Myers Squibb, Merck Sharp & Dohme, Roche-Genenetech. He received research fund from Bristol Myers Squibb.

AMG has no competing interest.

NA has no competing interest.

LB has no competing interest.

LC has no competing interest.

NC has no competing interest.

AC is full time employee of Nodality Inc., salary and shares.

MDV received Research Funding from Celgene, Novartis, Roche, and Glaxo SmithKline.

AME participated to advisory boards for BMS, GSK, MedImmune, MSD.

MF participated to advisory board for Amgen; he was also consultant for Delcath.

SF has no competing interest.

BAF is a co-founder of and has an ownership interest in UbiVac, the company developing autophagosome vaccine technology.

TG participated to advisory board from GSK-Bio, Roche-Genentech, Merck, BMS, Abbvie; he is co-founder of Jounce; he received research support from GSK-Bio, Eisai, Roche-Genentech, BMS, Curetech, Morphotek, Incyte.

JG is consulting for Ventana-Roche; he participated to advisory board for Jounce; he has a research contract with MedImmune.

SG is an inventor on patents/patent application regarding the NY-ESO-1 antigen, and Ludwig Institute for Cancer Research has licensed such patents.

HG has an advisory role for Roche, Glaxo, BMS, and MSD.

MKS owns stock in Melanoma Diagnostics, Inc.

HLK has research support, participated to advisory board and received honoraria from Amgen.

JL participated to advisory boards from Pfizer, Novartis, Aveo, BMS, GSK; he received research funding from Novartis and Pfizer.

RL has no competing interest.

AM has no competing interest.

KM received clinical research support from Genentech/Roche, BMS, GSK, Merck, Altor, Novartis, Prometheus.

CM is CSO of ISA Pharmaceuticals, trying to market SLP vaccines, for which he receives a salary and has stock appreciation rights.

GMA has research support from Pfizer, Novartis and Millennium.

GP has no competing interest.

IP has no competing interest.

AR has been a consultant for Amgen, GSK, Roche, Novartis with the honoraria paid to UCLA.

BS has no competing interest.

JS participated to advisory board for BMS, GSK, Roche-Genentech with honorarium.

PS is an employee of GSK Vaccines.

AAT has participated to advisory board from Merck, Genentech, Prometheus, AstraZeneca; he received research funding (contracted with the University of Pittsburgh) from Merck, BMS, Prometheus, Pfizer, Novartis, Amgen.

GT has no competing interest.

FVV has no competing interest.

EW has no competing interest.

GC has no competing interest.

NM has no competing interest.

FMM has no competing interest.

MT has no competing interest.

## Authors’ contributions

PAA, AMG, and MT prepared the manuscript collaboratively with input and review by all co-authors. All authors read and approved the final manuscript.

All the data and results reported into the manuscript are compliant with the Helsinki Declaration and the international guidelines regarding any experimental research.

## References

[B1] Clark WH Jr From L Bernardino EA Mihm MC The histogenesis and biologic behavior of primary human malignant melanomas of the skin Cancer Res 1969 29 705 727 5773814

[B2] Korn EL Liu PY Lee SJ Chapman JA Niedzwiecki D Suman VJ Moon J Sondak VK Atkins MB Eisenhauer EA Parulekar W Markovic SN Saxman S Kirkwood JM Meta-analysis of phase II cooperative group trials in metastatic stage IV melanoma to determine progression-free and overall survival benchmarks for future phase II trials J Clin Oncol 2008 26 527 534 10.1200/JCO.2007.12.7837 18235113

[B3] Flaherty KT Puzanov I Kim KB Ribas A McArthur GA Sosman JA O’Dwyer PJ Lee RJ Grippo JF Nolop K Chapman PB Inhibition of mutated, activated BRAF in metastatic melanoma N Engl J Med 2010 363 809 819 10.1056/NEJMoa1002011 20818844 PMC3724529

[B4] Falchook GS Long GV Kurzrock R Kim KB Arkenau TH Brown MP Hamid O Infante JR Millward M Pavlick AC O’Day SJ Blackman SC Curtis CM Lebowitz P Ma B Ouellet D Kefford RF Dabrafenib in patients with melanoma, untreated brain metastases, and other solid tumours: a phase 1 dose-escalation trial Lancet 2012 379 1893 1901 10.1016/S0140-6736(12)60398-5 22608338 PMC4109288

[B5] Van den Eynde BJ van der Bruggen P T cell defined tumor antigens Curr Opin Immunol 1997 9 684 693 10.1016/S0952-7915(97)80050-7 9368778

[B6] Gérard C Baudson N Ory T Piccininno R Brichard V Comprehensive preclinical model evaluating a protein-based MAGE-A3 specific cancer immunotherapy to fight against MAGE-A3 expressing tumors [abstract] Eur J Canc 2006 4 39

[B7] Kruit WH Suciu S Dreno B Chiarion-Sileni V Mortier L Robert C Maio M Brichard V Spatz A Eggermont A Keilholz U Lehmann F Immunization with the recombinant MAGE-A3 protein combined with Adjuvant Systems AS02B or AS15 in patients with metastatic melanoma: final results of a Phase II study of the EORTC Melanoma Group [abstract] Perspectives in Melanoma 2009 0 0001

[B8] Louahed J Lehmann F Ulloa-Montoya F Gruselle O Dizier B Vansteenkiste J Kruit W Brichard V Identification of a gene expression signature predictive of clinical activity following MAGE-A3 ASCI treatment [abstract] Mol Cancer Ther 2009 8 12 supplements 119139107

[B9] Yuan J Adamow M Ginsberg BA Rasalan TS Ritter E Gallardo HF Xu Y Pogoriler E Terzulli SL Kuk D Panageas KS Ritter G Sznol M Halaban R Jungbluth AA Allison JP Old LJ Wolchok JD Gnjatic S Integrated NY-ESO-1 antibody and CD8+ T-cell responses correlate with clinical benefit in advanced melanoma patients treated with ipilimumab Proc Natl Acad Sci USA 2011 108 16723 16728 10.1073/pnas.1110814108 21933959 PMC3189057

[B10] O’Donnell-Tormey J McDermott EA Jr The cancer vaccine collaborative: a new model of coordinated discovery Cancer Immun 2012 12 10 22896755 PMC3380350

[B11] Wheatley K Ives N Eggermont A Kirkwood J Cascinelli N Markovic SN Hancock B Lee S Suciu S on behalf of International Malignant Melanoma Collaborative Group Interferon-α as adjuvant therapy for melanoma: An individual patient data meta-analysis of randomised trials [abstract] Proc Am Soc Clin Oncol 2007 25 478S abstr 8526

[B12] Mocellin S Pasquali S Rossi CR Nitti D Interferon alpha adjuvant therapy in patients with high-risk melanoma: a systematic review and meta-analysis J Natl Cancer Inst 2010 102 493 501 10.1093/jnci/djq009 20179267

[B13] Moschos SJ Edington HD Land SR Rao UN Jukic D Shipe-Spotloe J Kirkwood JM Neoadjuvant treatment of regional stage IIIB melanoma with high-dose interferon alfa-2b induces objective tumor regression in association with modulation of tumor infiltrating host cellular immune responses J Clin Oncol 2006 24 3164 3171 10.1200/JCO.2005.05.2498 16809739

[B14] Wang W Edington HD Rao UN Jukic DM Land SR Ferrone S Kirkwood JM Modulation of signal transducers and activators of transcription 1 and 3 signaling in melanoma by high-dose IFNalpha2b Clin Cancer Res 2007 13 1523 1531 10.1158/1078-0432.CCR-06-1387 17332298

[B15] Yurkovetsky ZR Kirkwood JM Edington HD Marrangoni AM Velikokhatnaya L Winans MT Gorelik E Lokshin AE Multiplex analysis of serum cytokines in melanoma patients treated with interferon-alpha2b Clin Cancer Res 2007 13 2422 2428 10.1158/1078-0432.CCR-06-1805 17438101

[B16] Meyer S Wild PJ Vogt T Bataille F Ehret C Gantner S Landthaler M Klinkhammer-Schalke M Hofstaedter F Bosserhoff AK Methylthioadenosine phosphorylase represents a predictive marker for response to adjuvant interferon therapy in patients with malignant melanoma Exp Dermatol 2010 19 e251 e257 20500769 10.1111/j.1600-0625.2010.01072.x

[B17] Wang E Zhao Y Monaco A Uccellini L Kirkwood JM Spyropoulou-Vlachou M Panelli MC Marincola FM Gogas H A multi-factorial genetic model for prognostic assessment of high risk melanoma patients receiving adjuvant interferon PLoS One 2012 7 e40805 10.1371/journal.pone.0040805 22911710 PMC3404079

[B18] Gogas H Kirkwood JM Falk CS Dafni U Sondak VK Tsoutsos D Stratigos A Markopoulos C Pectasides D Spyropoulou-Vlachou M Correlation of molecular human leukocyte antigen typing and outcome in high-risk melanoma patients receiving adjuvant interferon Cancer 2010 116 4326 4333 10.1002/cncr.25211 20549830 PMC2970916

[B19] Gogas H Dafni U Koon H Spyropoulou-Vlachou M Metaxas Y Buchbinder E Pectasides E Tsoutsos D Polyzos A Stratigos A Markopoulos C Panagiotou P Fountzilas G Castana O Skarlos P Atkins MB Kirkwood JM Evaluation of six CTLA-4 polymorphisms in high-risk melanoma patients receiving adjuvant interferon therapy in the He13A/98 multicenter trial J Transl Med 2010 8 108 10.1186/1479-5876-8-108 21044351 PMC2988721

[B20] Wang W Yu D Sarnaik AA Yu B Hall M Morelli D Zhang Y Zhao X Weber JS Biomarkers on melanoma patient T cells associated with ipilimumab treatment J Transl Med 2012 10 146 10.1186/1479-5876-10-146 22788688 PMC3527361

[B21] Irish JM Hovland R Krutzik PO Perez OD Bruserud Ø Gjertsen BT Nolan GP Single cell profiling of potentiated phospho-protein networks in cancer cells Cell 2004 118 217 228 10.1016/j.cell.2004.06.028 15260991

[B22] Cesano A Rosen DB O’Meara P Putta S Gayko U Spellmeyer DC Cripe LD Sun Z Uno H Litzow MR Tallman MS Paietta E Functional pathway analysis in acute myeloid leukemia using single cell network profiling assay: effect of specimen source (bone marrow or peripheral blood) on assay readouts Cytometry B Clin Cytom 2012 82 158 172 22334473 10.1002/cyto.b.21007 PMC3632455

[B23] Covey TM Cesano A Modulated multiparametric phosphoflow cytometry in hematological malignancies: technology and clinical applications Best Pract Res Clin Haematol 2010 23 319 331 10.1016/j.beha.2010.07.002 21112033

[B24] Covey TM Cesano A Parkinson DR Single-cell network profiling (SCNP) by flow cytometry in autoimmune disease Autoimmunity 2010 43 550 559 10.3109/08916931003674774 20482423

[B25] Longo DM Louie B Putta S Evensen E Ptacek J Cordeiro J Wang E Pos Z Hawtin RE Marincola FM Cesano A Single-cell network profiling of peripheral blood mononuclear cells from healthy donors reveals age- and race-associated differences in immune signaling pathway activation J Immunol 2012 188 1717 1725 10.4049/jimmunol.1102514 22246624 PMC3517183

[B26] Hotson D Conroy A Evensen E Gentilcore G Simeone E Esposito A Curvietto M Cesano A Hawtin R Ascierto PA CTLA-4 defines distinct T cell signaling populations in healthy donors and metastatic melanoma patients [abstract] J Immunother 2012 35 suppl. 9 760

[B27] Kashani-Sabet M Venna S Nosrati M Rangel J Sucker A Egberts F Baehner FL Simko J Leong SP Haqq C Hauschild A Schadendorf D Miller JR 3rd Sagebiel RW A multimarker prognostic assay for primary cutaneous melanoma Clin Cancer Res 2009 15 6987 6992 10.1158/1078-0432.CCR-09-1777 19887476 PMC2784204

[B28] De Semir D Nosrati M Bezrookove V Dar AA Federman S Bienvenu G Venna S Rangel J Climent J Meyer Tamgüney TM Thummala S Tong S Leong SP Haqq C Billings P Miller JR 3rd Sagebiel RW Debs R Kashani-Sabet M Pleckstrin homology domain-interacting protein (PHIP) as a marker and mediator of melanoma metastasis Proc Natl Acad Sci USA 2012 109 7067 7072 10.1073/pnas.1119949109 22511720 PMC3344978

[B29] Kirkwood JM Ibrahim JG Sosman JA Sondak VK Agarwala SS Ernstoff MS Rao U High-dose interferon alfa-2b significantly prolongs relapse-free and overall survival compared with the GM2-KLH/QS-21 vaccine in patients with resected stage IIB-III melanoma: results of intergroup trial E1694/S9512/C509801 J Clin Oncol 2001 19 2370 2380 11331315 10.1200/JCO.2001.19.9.2370

[B30] Eggermont AM Suciu S Santinami M Kruit W Testori A Marsden J Punt CJA Gore ME MacKie R Dummer R Schadendorf D Patel P Spatz A Keilholz U EORTC 18991 phase III trial: Long-term adjuvant pegylated interferon-α2b (PEG-IFN) versus observation in resected stage III melanoma: long-term results at 7.6-years follow-up [abstract] J Clin Oncol 2011 29 abstr 8506b

[B31] Gogas H Ioannovich J Dafni U Stavropoulou-Giokas C Frangia K Tsoutsos D Panagiotou P Polyzos A Papadopoulos O Stratigos A Markopoulos C Bafaloukos D Pectasides D Fountzilas G Kirkwood JM Prognostic significance of autoimmunity during treatment of melanoma with interferon N Engl J Med 2006 354 709 718 10.1056/NEJMoa053007 16481638

[B32] Ascierto PA Kirkwood JM Adjuvant therapy of melanoma with interferon: lessons of the past decade J Transl Med 2008 6 62 10.1186/1479-5876-6-62 18954464 PMC2605741

[B33] Kumar KG Liu J Li Y Yu D Thomas-Tikhonenko A Herlyn M Fuchs SY Raf inhibitor stabilizes receptor for the type I interferon but inhibits its anti-proliferative effects in human malignantmelanoma cells Cancer Biol Ther 2007 6 1437 1441 10.4161/cbt.6.9.4569 17873516 PMC2254442

[B34] Tarhini AA Cherian J Moschos SJ Tawbi HA Shuai Y Gooding WE Sander C Kirkwood JM Safety and efficacy of combination immunotherapy with interferon alfa-2b and tremelimumab in patients with stage IV melanoma J Clin Oncol 2012 30 322 328 10.1200/JCO.2011.37.5394 22184371 PMC3422533

[B35] Peng W Liu C Xu C Lou Y Chen J Yang Y Yagita H Overwijk WW Lizée G Radvanyi L Hwu P PD-1 blockade enhances T-cell migration to tumors by elevating IFN-γ inducible chemokines Cancer Res 2012 72 5209 5218 10.1158/0008-5472.CAN-12-1187 22915761 PMC3476734

[B36] Lawson DH Lee SJ Tahrini AA Margolin KK Ernstoff MS Kirkwood JM E4697: Phase III cooperative group study of yeast-derived granulocyte macrophage colony-stimulating factor (GM-CSF) versus placebo as adjuvant treatment of patients with completed resected stage III-IV melanoma J Clin Oncol 2010 28 suppl 8507 abstract

[B37] Flaherty LE Moon J Atkins MB Tuthill R Thompson JA Vetto JT Haluska FG Pappo AS Sosman JA Redman BG Ribas A Kirkwood JM Sondak VK Phase III trial of high-dose interferon alpha-2b versus cisplatin, vinblastine, DTIC plus IL-2 and interferon in patients with high-risk melanoma (SWOG S0008): an intergroup study of CALGB, COG, ECOG, and SWOG [abstract] J Clin Oncol 2012 30 8504 abstr

[B38] Overwijk WW Tsung A Irvine KR Parkhurst MR Goletz TJ Tsung K Carroll MW Liu C Moss B Rosenberg SA Restifo NP gp100/pmel 17 is a murine tumor rejection antigen: induction of “self”-reactive, tumoricidal T cells using high-affinity, altered peptide ligand J Exp Med 1998 188 277 286 10.1084/jem.188.2.277 9670040 PMC2212458

[B39] Hodis E Watson IR Kryukov GV Arold ST Imielinski M Theurillat JP Nickerson E Auclair D Li L Place C Dicara D Ramos AH Lawrence MS Cibulskis K Sivachenko A Voet D Saksena G Stransky N Onofrio RC Winckler W Ardlie K Wagle N Wargo J Chong K Morton DL Stemke-Hale K Chen G Noble M Meyerson M Ladbury JE A landscape of driver mutations in melanoma Cell 2012 150 2 251 263 10.1016/j.cell.2012.06.024 22817889 PMC3600117

[B40] Davies MA Samuels Y Analysis of the genome to personalize therapy for melanoma Oncogene 2010 29 5545 5555 10.1038/onc.2010.323 20697348 PMC3169242

[B41] Dankort D Curley DP Cartlidge RA Nelson B Karnezis AN Damsky WE Jr You MJ DePinho RA McMahon M Bosenberg M Braf (V600E) cooperates with Pten loss to induce metastatic melanoma Nat Genet 2009 41 544 552 10.1038/ng.356 19282848 PMC2705918

[B42] Rosenberg SA Yang JC Sherry RM Kammula US Hughes MS Phan GQ Citrin DE Restifo NP Robbins PF Wunderlich JR Morton KE Laurencot CM Steinberg SM White DE Dudley ME Durable complete responses in heavily pretreated patients with metastatic melanoma using T-cell transfer immunotherapy Clin Cancer Res 2011 17 4550 4557 10.1158/1078-0432.CCR-11-0116 21498393 PMC3131487

[B43] Boni A Cogdill AP Dang P Udayakumar D Njauw CN Sloss CM Ferrone CR Flaherty KT Lawrence DP Fisher DE Tsao H Wargo JA Selective BRAF V600E inhibition enhances T-cell recognition of melanoma without affecting lymphocyte function Cancer Res 2010 70 13 5213 5219 10.1158/0008-5472.CAN-10-0118 20551059

[B44] Cartlidge RA Thomas GR Cagnol S Jong KA Molton SA Finch AJ McMahon M Oncogenic BRAF (V600E) inhibits BIM expression to promote melanoma cell survival Pigment Cell Melanoma Res 2008 21 534 544 10.1111/j.1755-148X.2008.00491.x 18715233 PMC4916913

[B45] Boisvert-Adamo K Aplin AE Mutant B-RAF mediates resistance to anoikis via Bad and Bim Oncogene 2008 27 3301 3312 10.1038/sj.onc.1211003 18246127

[B46] Sumimoto H Imabayashi F Iwata T Kawakami Y The BRAF-MAPK signaling pathway is essential for cancer-immune evasion in human melanoma cells J Exp Med 2006 203 1651 1656 10.1084/jem.20051848 16801397 PMC2118331

[B47] Chapman PB Hauschild A Robert C Haanen JB Ascierto P Larkin J Dummer R Garbe C Testori A Maio M Hogg D Lorigan P Lebbe C Jouary T Schadendorf D Ribas A O’Day SJ Sosman JA Kirkwood JM Eggermont AM Dreno B Nolop K Li J Nelson B Hou J Lee RJ Flaherty KT McArthur GA BRIM-3 Study Group Improved survival with vemurafenib in melanoma with BRAF V600E mutation New England J Med 2011 364 2507 2516 10.1056/NEJMoa1103782 21639808 PMC3549296

[B48] Kopetz S Desai J Chan E Hecht JR O’Dwyer PJ Lee RJ Nolop KB Saltz L PLX4032 in metastatic colorectal cancer patients with mutant BRAF tumors [abstract] J Clin Oncol 2010 28 abstract 3534

[B49] Prahallad A Sun C Huang S Di Nicolantonio F Salazar R Zecchin D Beijersbergen RL Bardelli A Bernards R Unresponsiveness of colon cancer to BRAF(V600E) inhibition through feedback activation of EGFR Nature 2012 483 100 104 10.1038/nature10868 22281684

[B50] Kim K Cabanillas M Lazar AJ Williams MD Sanders DL Ilagan JL Nolop K Lee RJ Sherman SI Clinical Responses to Vemurafenib in Patients with Metastatic Papillary Thyroid Cancer Harboring V600EBRAF Mutation Thyroid 2013 [Epub ahead of print] PubMed PMID: 23489023 10.1089/thy.2013.0057 PMC3967415 23489023

[B51] A Study of Vemurafenib in Patients With BRAF V600 Mutation-Positive Cancers (NCT01524978) http://www.clinicaltrials.gov

[B52] Yang H Higgins B Kolinsky K Packman K Go Z Iyer R Kolis S Zhao S Lee R Grippo JF Schostack K Simcox ME Heimbrook D Bollag G Su F RG7204 (PLX4032), a selective BRAFV600E inhibitor, displays potent antitumor activity in preclinical melanoma models Cancer Res 2010 70 5518 5527 10.1158/0008-5472.CAN-10-0646 20551065

[B53] Sullivan RJ Lawrence DP Flaherty KT McDermott DF Aldridge J Cho DC Simonson R Seery VJ Hodi FS Atkins MB Mier JW Panka DJ Predicting early relapse in patients with BRAFV600E melanoma with a highly sensitive blood BRAF assay [abstract] J Clin Oncol 2012 30 abstr 8516

[B54] Hauschild A Grob JJ Demidov LV Jouary T Gutzmer R Millward M Rutkowski P Blank CU Miller WH Jr Kaempgen E Martín-Algarra S Karaszewska B Mauch C Chiarion-Sileni V Martin AM Swann S Haney P Mirakhur B Guckert ME Goodman V Chapman PB Dabrafenib in BRAF-mutated metastatic melanoma: a multicentre, open-label, phase 3 randomised controlled trial Lancet 2012 380 358 365 10.1016/S0140-6736(12)60868-X 22735384

[B55] Gonzalez R Ribas A Daud A Pavlick A Gajewski TF Puzanov I Teng MSL Chan I Choong NW McArthur G Phase Ib study of vemurafenib in combination with the MEK inhibitor, GDC-0973, in patients (pts) with unresectable or metastatic BRAFV600 mutated melanoma (BRIM7) [abstract] Ann Oncol 2012 23 ixe20

[B56] Tiano HF Loftin CD Akunda J Lee CA Spalding J Sessoms A Dunson DB Rogan EG Morham SG Smart RC Langenbach R Deficiency of either cyclooxygenase (COX)-1 or COX-2 alters epidermal differentiation and reduces mouse skin tumorigenesis Cancer Res 2002 62 3395 3401 12067981

[B57] Trunzer K Pavlick AC Schuchter L Gonzalez R McArthur GA Hutson TE Moschos SJ Flaherty KT Kim KB Weber JS Hersey P Long GV Lawrence D Ott PA Amaravadi RK Lewis KD Puzanov I Lo RS Koehler A Kockx M Spleiss O Schell-Steven A Gilbert HN Cockey L Bollag G Lee RJ Joe AK Sosman JA Ribas A Pharmacodynamic Effects and Mechanisms of Resistance to Vemurafenib in Patients With Metastatic Melanoma J Clin Oncol 2013 Epub ahead of print 10.1200/JCO.2012.44.7888 23569304

[B58] Nazarian R Shi H Wang Q Kong X Koya RC Lee H Chen Z Lee MK Attar N Sazegar H Chodon T Nelson SF McArthur G Sosman JA Ribas A Lo RS Melanomas acquire resistance to B-RAF(V600E) inhibition by RTK or N-RAS upregulation Nature 2010 468 973 977 10.1038/nature09626 21107323 PMC3143360

[B59] Whittaker SR Theurillat JP Van Allen E Wagle N Hsiao J Cowley GS Schadendorf D Root DE Garraway LA A genome-scale RNA interference screen implicates NF1 loss in resistance to RAF inhibition Cancer Discov 2013 3 350 362 10.1158/2159-8290.CD-12-0470 23288408 PMC3606893

[B60] Maertens O Johnson B Hollstein P Frederick DT Cooper ZA Messiaen L Bronson RT McMahon M Granter S Flaherty K Wargo JA Marais R Cichowski K Elucidating distinct roles for NF1 in melanomagenesis Cancer Discov 2013 3 338 349 10.1158/2159-8290.CD-12-0313 23171796 PMC3595355

[B61] Chiocca EA Oncolytic viruses Nat Rev Cancer 2002 2 938 950 10.1038/nrc948 12459732

[B62] Kudo-Saito C Schlom J Hodge JW Induction of an antigen cascade by diversified subcutaneous/intratumoral vaccination is associated with antitumor responses Clin Cancer Res 2005 11 2416 2426 10.1158/1078-0432.CCR-04-1380 15788693

[B63] Senzer NN Kaufman HL Amatruda T Nemunaitis M Reid T Daniels G Gonzalez R Glaspy J Whitman E Harrington K Goldsweig H Marshall T Love C Coffin R Nemunaitis JJ Phase II clinical trial of a granulocyte-macrophage colony-stimulating factor–encoding, second-generation oncolytic herpesvirus in patients with unresectable metastatic melanoma J Clin Oncol 2009 27 5763 5771 10.1200/JCO.2009.24.3675 19884534

[B64] Trinchieri G Gut commensal bacteria determine cancer response to treatment by modulating systemic inflammation [abstract] Ann Oncol 2013 24 i12

[B65] Trinchieri G Cancer and Inflammation: An old intuition with rapidly evolving new concepts Annual Rev Immunology 2012 30 677 706 10.1146/annurev-immunol-020711-075008 22224761

[B66] Cataisson C Salcedo R Hakim S Moffitt BA Wright L Yi M Stephens R Dai RM Lyakh L Schenten D Yuspa HS Trinchieri G IL-1R-MyD88 signaling in keratinocyte transformation and carcinogenesis J Exp Med 2012 209 1689 1702 10.1084/jem.20101355 22908325 PMC3428947

[B67] Mantovani A Allavena P Sica A Balkwill F Cancer-related inflammation Nature 2008 454 436 444 10.1038/nature07205 18650914

[B68] Sica A Mantovani A Macrophage plasticity and polarization: *in vivo* veritas J Clin Invest 2012 122 787 795 10.1172/JCI59643 22378047 PMC3287223

[B69] Mantovani A Romero P Palucka AK Marincola FM Tumour immunity: effector response to tumour and role of the microenvironment Lancet 2008 371 771 783 10.1016/S0140-6736(08)60241-X 18275997

[B70] Biswas SK Allavena P Mantovani A Tumor-associated macrophages: functional diversity, clinical significance and open questions Semin Immunopathology 2013 in press 10.1007/s00281-013-0367-7 23657835

[B71] Bottazzi B Doni A Garlanda C Mantovani A An integrated view of humoral innate immunity: pentraxins as a paradigm Annu Rev Immunol 2010 28 157 183 10.1146/annurev-immunol-030409-101305 19968561

[B72] Beatty GL Chiorean EG Fishman MP Saboury B Teitelbaum UR Sun W Huhn RD Song W Li D Sharp LL Torigian DA O’Dwyer PJ Vonderheide RH CD40 agonists alter tumor stroma and show efficacy against pancreatic carcinoma in mice and humans Science 2011 331 1612 1616 10.1126/science.1198443 21436454 PMC3406187

[B73] Germano G Frapolli R Belgiovine C Anselmo A Pesce S Liguori M Erba E Uboldi S Zucchetti M Pasqualini F Nebuloni M van Rooijen N Mortarini R Beltrame L Marchini S Fuso Nerini I Sanfilippo R Casali PG Pilotti S Galmarini CM Anichini A Mantovani A D'Incalci M Allavena P Role of macrophage targeting in the anti-tumor activity of Trabectedin Cancer Cell 2013 23 249 262 10.1016/j.ccr.2013.01.008 23410977

[B74] Balch CM Gershenwald JE Soong SJ Thompson JF Atkins MB Byrd DR Buzaid AC Cochran AJ Coit DG Ding S Eggermont AM Flaherty KT Gimotty PA Kirkwood JM McMasters KM Mihm MC Jr Morton DL Ross MI Sober AJ Sondak VK Final version of 2009 AJCC melanoma staging and classification J Clin Oncol 2009 27 6199 6206 10.1200/JCO.2009.23.4799 19917835 PMC2793035

[B75] Spatz A Cook MG Elder DE Piepkorn M Ruiter DJ Barnhill RL Interobserver reproducibility of ulceration assessment in primary cutaneous melanomas Eur J Cancer 2003 39 1861 1865 10.1016/S0959-8049(03)00325-3 12932663

[B76] Winnepenninckx V Lazar V Michiels S Dessen P Stas M Alonso SR Avril MF Ortiz Romero PL Robert T Balacescu O Eggermont AM Lenoir G Sarasin A Tursz T van den Oord JJ Spatz A Melanoma Group of the European Organization for Research and Treatment of Cancer Gene expression profiling of primary cutaneous melanoma and clinical outcome J Natl Cancer Inst 2006 98 472 482 10.1093/jnci/djj103 16595783

[B77] Elliott B Scolyer RA Suciu S Lebecque S Rimoldi D Gugerli O Musat E Sharma RN Lienard D Keilholz U Testori A Eggermont A MacKie R Robert C Cook M Thompson JF Angevin E Spatz A European Organization for Research and Treatment of Cancer Melanoma Group Long-term protective effect of mature DC-LAMP + dendritic cell accumulation in sentinel lymph nodes containing micrometastatic melanoma Clin Cancer Res 2007 13 3825 3830 10.1158/1078-0432.CCR-07-0358 17606713

[B78] Eggermont AM Suciu S MacKie R Ruka W Testori A Kruit W Punt CJ Delauney M Sales F Groenewegen G Ruiter DJ Jagiello I Stoitchkov K Keilholz U Lienard D EORTC Melanoma Group Post-surgery adjuvant therapy with intermediate doses of interferon alfa 2b versus observation in patients with stage IIb/III melanoma (EORTC 18952): randomised controlled trial Lancet 2005 366 1189 1196 10.1016/S0140-6736(05)67482-X 16198768

[B79] Eggermont AM Suciu S Santinami M Testori A Kruit WH Marsden J Punt CJ Salès F Gore M Mackie R Kusic Z Dummer R Hauschild A Musat E Spatz A Keilholz U EORTC Melanoma Group Adjuvant therapy with pegylated interferon alfa-2b versus observation alone in resected stage III melanoma: final results of EORTC 18991, a randomised phase III trial Lancet 2008 372 117 126 10.1016/S0140-6736(08)61033-8 18620949

[B80] Eggermont AM Suciu S Testori A Kruit WH Marsden J Punt CJ Santinami M Salès F Schadendorf D Patel P Dummer R Robert C Keilholz U Yver A Spatz A Ulceration and stage are predictive of interferon efficacy in melanoma: Results of the phase III adjuvant trials EORTC 18952 and EORTC 18991 Eur J Cancer 2012 48 218 225 10.1016/j.ejca.2011.09.028 22056637

[B81] Eggermont AM Suciu S Testori A Santinami M Kruit WH Marsden J Punt CJ Salès F Dummer R Robert C Schadendorf D Patel PM de Schaetzen G Spatz A Keilholz U Long-term results of the randomized phase III trial EORTC 18991 of adjuvant therapy with pegylated interferon alfa-2b versus observation in resected stage III melanoma J Clin Oncol 2012 30 31 3810 3818 10.1200/JCO.2011.41.3799 23008300

[B82] McMasters KM Edwards MJ Ross MI Reintgen DS Martin RC 2nd Urist MM Noyes RD Sussman JJ Stromberg AJ Scoggins CR Ulceration as a predictive marker for response to adjuvant interferon therapy in melanoma Ann Surg 2010 252 460 465 20739846 10.1097/SLA.0b013e3181f20bb1

[B83] Gerlinger M Rowan AJ Horswell S Larkin J Endesfelder D Gronroos E Martinez P Matthews N Stewart A Tarpey P Varela I Phillimore B Begum S McDonald NQ Butler A Jones D Raine K Latimer C Santos CR Nohadani M Eklund AC Spencer-Dene B Clark G Pickering L Stamp G Gore M Szallasi Z Downward J Futreal PA Swanton C Intratumor heterogeneity and branched evolution revealed by multiregion sequencing N Engl J Med 2012 366 883 892 10.1056/NEJMoa1113205 22397650 PMC4878653

[B84] Seghers AC Wilgenhof S Lebbé C Neyns B Successful rechallenge in two patients with BRAF-V600-mutant melanoma who experienced previous progression during treatment with a selective BRAF inhibitor Melanoma Res 2012 22 466 472 10.1097/CMR.0b013e3283541541 22584957

[B85] Koay E Sulman EP Management of brain metastasis: past lessons, modern management, and future considerations Curr Oncol Rep 2012 14 70 78 10.1007/s11912-011-0205-9 22071681

[B86] Rochet NM Kottschade LA Markovic SN Vemurafenib for melanoma metastases to the brain N Engl J Med 2011 365 2439 2441 10.1056/NEJMc1111672 22188003

[B87] Engelhardt B Ransohoff RM The ins and outs of T-lymphocyte trafficking to the CNS: anatomical sites and molecular mechanisms Trends Immunol 2005 26 485 495 10.1016/j.it.2005.07.004 16039904

[B88] Davies MA Stemke-Hale K Lin E Tellez C Deng W Gopal YN Woodman SE Calderone TC Ju Z Lazar AJ Prieto VG Aldape K Mills GB Gershenwald JE Integrated Molecular and Clinical Analysis of AKT Activation in Metastatic Melanoma Clin Cancer Res 2009 15 7538 7546 10.1158/1078-0432.CCR-09-1985 19996208 PMC2805170

[B89] Desai N Trieu V Yao Z Louie L Ci S Yang A Tao C De T Beals B Dykes D Noker P Yao R Labao E Hawkins M Soon-Shiong P Increased antitumor activity, intratumor paclitaxel concentrations, and endothelial cell transport of cremophor-free, albumin-bound paclitaxel, ABI-007, compared with cremophor-based paclitaxel Clin Cancer Res 2006 12 1317 1324 10.1158/1078-0432.CCR-05-1634 16489089

[B90] Hersh E Del Vecchio M Brown M Kefford R Loquai C Testori A Bhatia S Gutzmer R Haydon A Robert C Clawson A Elias I Renschler M Hauschild A Phase 3, randomized, open-label, multicenter trial of nab-paclitaxel (nab-P) versus dacarbazine (DTIC) in previously untreated patients with metastatic malignant melanoma (MMM) [abstract] Pigment Cell Melanoma Res 2012 25 863

[B91] Gattinoni L Lugli E Ji Y Pos Z Paulos CM Quigley MF Almeida JR Gostick E Yu Z Carpenito C Wang E Douek DC Price DA June CH Marincola FM Roederer M Restifo NP A human memory T cell subset with stem cell-like properties Nat Med 2011 17 1290 1297 10.1038/nm.2446 21926977 PMC3192229

[B92] Uccellini L De Giorgi V Zhao Y Tumaini B Erdenebileg N Dudley ME Tomei S Bedognetti D Ascierto ML Liu Q Simon R Kottyan L Kaufman KM Harley JB Wang E Rosenberg SA Marincola FM IRF5 gene polymorphisms in melanoma J Transl Med 2012 10 170 10.1186/1479-5876-10-170 22909381 PMC3492128

[B93] Wendler T Herrmann K Schnelzer A Lasser T Traub J Kutter O Ehlerding A Scheidhauer K Schuster T Kiechle M Schwaiger M Ziegler N Buck AK First demonstration of 3-D lymphatic mapping in breast cancer using freehand SPECT Eur J Nucl Med Mol Imaging 2010 37 1452 1461 10.1007/s00259-010-1430-4 20354851

[B94] Gerlini G Urso C Mariotti G Di Gennaro P Palli D Brandani P Salvadori A Pimpinelli N Reali UM Borgognoni L Plasmacytoid dendritic cells represent a major dendritic cell subset in sentinel lymph nodes of melanoma patients and accumulate in metastatic nodes Clin Immunol 2007 125 184 193 10.1016/j.clim.2007.07.018 17827069

[B95] Gerlini G Di Gennaro P Mariotti G Urso C Chiarugi A Caporale R Pimpinelli N Borgognoni L Human Langerhans cells are immature in melanoma sentinel lymph nodes Blood 2012 119 4807 4808 10.1182/blood-2011-12-401067 22596171

[B96] Gerlini G Sestini S Di Gennaro P Urso C Pimpinelli N Borgognoni L Dendritic cells recruitment in melanoma metastasis treated by electrochemotherapy Clin Exp Metastasis 2013 30 37 45 10.1007/s10585-012-9505-1 22735940

[B97] Gerlini G Di Gennaro P Borgognoni L Enhancing anti-melanoma immunity by electrochemotherapy and *in vivo* dendritic-cell activation Oncoimmunology 2012 1 1655 1657 10.4161/onci.21991 23264927 PMC3525636

[B98] Pingpank JF Libutti SK Chang R Wood BJ Neeman Z Kam AW Figg WD Zhai S Beresneva T Seidel GD Alexander HR Phase I study of hepatic arterial melphalan infusion and hepatic venous hemofiltration using percutaneouslyplaced catheters in patients with unresectable hepatic malignancies J Clin Oncol 2005 23 3465 3474 10.1200/JCO.2005.00.927 15908655 PMC2374756

[B99] Pingpank JF Hughes MS Faries MB Zager JS Alexander HR Royal R Whitman ED Nutting CW Siskin GP Agarwala SS A phase III random assignment trial comparing percutaneous hepatic perfusion with melphalan (PHP-mel) to standard of care for patients with hepatic metastases from metastatic ocular or cutaneous melanoma [abstract] J Clin Oncol 2010 28 18s abstr. LBA8512

[B100] Seliger B Novel insights into molecular mechanisms of HLA class I abnormalities Cancer Immunol Immunother 2012 61 249 254 10.1007/s00262-011-1153-9 22120755 PMC11029063

[B101] Amiot L Ferrone S Grosse-Wilde H Seliger B Biology of HLA-G in cancer: a candidate molecule for therapeutic intervention? Cell Mol Life Sci 2011 68 417 431 10.1007/s00018-010-0583-4 21063893 PMC3426238

[B102] Nemunaitis J Sterman D Jablons D Smith JW 2nd Fox B Maples P Hamilton S Borellini F Lin A Morali S Hege K Granulocyte-macrophage colony-stimulating factor gene-modified autologous tumor vaccines in non-small-cell lung cancer J Natl Cancer Inst 2004 96 326 331 10.1093/jnci/djh028 14970281

[B103] Twitty CG Jensen SM Hu HM Fox BA Tumor-derived autophagosome vaccine: induction of cross-protective immuneresponses against short-lived proteins through a p62-dependent mechanism Clin Cancer Res 2011 17 6467 6481 10.1158/1078-0432.CCR-11-0812 21810919 PMC3298078

[B104] Kenter GG Welters MJ Valentijn AR Lowik MJ der Meer DM B-v Vloon AP Essahsah F Fathers LM Offringa R Drijfhout JW Wafelman AR Oostendorp J Fleuren GJ van der Burg SH Melief CJ Vaccination against HPV-16 oncoproteins for vulvar intraepithelial neoplasia N Engl J Med 2009 361 1838 1847 10.1056/NEJMoa0810097 19890126

[B105] Welters MJ Kenter GG DeVosvanSteenwijk PJ Löwik MJ Berends-van der Meer DM Essahsah F Stynenbosch LF Vloon AP Ramwadhdoebe TH Piersma SJ van der Hulst JM Valentijn AR Fathers LM Drijfhout JW Franken KL Oostendorp J Fleuren GJ Melief CJ van der Burg SH Success or failure of vaccination for HPV16-positive vulvar lesions correlates with kinetics and phenotype of induced T-cell responses Proc Natl Acad Sci USA 2010 107 11895 11899 10.1073/pnas.1006500107 20547850 PMC2900675

[B106] Calabrò L Danielli R Sigalotti L Maio M Clinical studies with anti-CTLA-4 antibodies in non-melanoma indications Semin Oncol 2010 37 460 467 10.1053/j.seminoncol.2010.09.006 21074061

[B107] Vidal-Vanaclocha F Mendoza L Telleria N Salado C Valcárcel M Gallot N Carrascal T Egilegor E Beaskoetxea J Dinarello CA Clinical and experimental approaches to the pathophysiology of interleukin-18 in cancer progression Cancer Metastasis Rev 2006 25 417 434 10.1007/s10555-006-9013-3 17001512

[B108] Vidal-Vanaclocha F Fantuzzi G Mendoza L Fuentes AM Anasagasti MJ Martin J Carrascal T Walsh P Reznikov LL Kim SH Novick D Rubinstein M Dinarello CA IL-18 regulates IL-1beta-dependent hepatic melanoma metastasis via vascular cell adhesion molecule-1 Proc Natl Acad Sci 2000 97 734 739 10.1073/pnas.97.2.734 10639148 PMC15399

[B109] Crende O Sabatino M Valcárcel M Carrascal T Riestra P López-Guerrero JA Nagore E Mandruzzato S Wang E Marincola FM Vidal-Vanaclocha F Metastatic lesions with interleukin-18 dependent and independent genes in advanced stage melanoma patients Am J Pathol 2013 in press 10.1016/j.ajpath.2013.03.026 23707237

[B110] Galon J Costes A Sanchez-Cabo F Kirilovsky A Mlecnik B Lagorce-Pagès C Tosolini M Camus M Berger A Wind P Zinzindohoué F Bruneval P Cugnenc PH Trajanoski Z Fridman WH Pagès F Type, density, and location of immune cells within human colorectal tumors predict clinical outcome Science 2006 313 1960 1964 10.1126/science.1129139 17008531

[B111] Fridman WH Pages F Sautes-Fridman C Galon J The immune contexture in human tumours: impact on clinical outcome Nat Rev Cancer 2012 12 298 306 10.1038/nrc3245 22419253

[B112] Mlecnik B Tosolini M Charoentong P Kirilovsky A Bindea G Berger A Camus M Gillard M Bruneval P Fridman WH Pagès F Trajanoski Z Galon J Biomolecular network reconstruction identifies T-cell homing factors associated with survival in colorectal cancer Gastroenterology 2010 138 1429 1440 10.1053/j.gastro.2009.10.057 19909745

[B113] Pagès F Kirilovsky A Mlecnik B Asslaber M Tosolini M Bindea G Lagorce C Wind P Marliot F Bruneval P Zatloukal K Trajanoski Z Berger A Fridman WH Galon J In situ cytotoxic and memory T cells predict outcome in patients with early-stage colorectal cancer J Clin Oncol 2009 27 5944 5951 10.1200/JCO.2008.19.6147 19858404

[B114] Mlecnik B Tosolini M Kirilovsky A Berger A Bindea G Meatchi T Bruneval P Trajanoski Z Fridman WH Pagès F Galon J Histopathologic-based prognostic factors of colorectal cancers are associated with the state of the local immune reaction J Clin Oncol 2011 29 610 618 10.1200/JCO.2010.30.5425 21245428

[B115] Galon J Pagès F Marincola FM Thurin M Trinchieri G Fox BA Gajewski TF Ascierto PA The immune score as a new possible approach for the classification of cancer J Transl Med 2012 10 1 10.1186/1479-5876-10-1 22214470 PMC3269368

[B116] Galon J Pagès F Marincola FM Angell HK Thurin M Lugli A Zlobec I Berger A Bifulco C Botti G Tatangelo F Britten CM Kreiter S Chouchane L Delrio P Arndt H Asslaber M Maio M Masucci GV Mihm M Vidal-Vanaclocha F Allison JP Gnjatic S Hakansson L Huber C Singh-Jasuja H Ottensmeier C Zwierzina H Laghi L Grizzi F Ohashi PS Cancer classification using the Immunoscore: a worldwide task force J Transl Med 2012 10 205 10.1186/1479-5876-10-205 23034130 PMC3554496

